# Multi-Scale Degradation Behavior and Durability of BF-WEP Modified Concrete Under Saline Environments

**DOI:** 10.3390/polym18192391

**Published:** 2026-09-30

**Authors:** Zhen Zhang, Lingtong Zhang, Liming Zhang, Fang Zhang, Yafei Chen, Baoyuan Li, Chuangzhou Wu

**Affiliations:** 1 School of Smart Water Conservancy Engineering, Xinjiang Institute of Technology, Aksu 843100, China; zz891010zz@163.com (Z.Z.); zhqling2024@163.com (L.Z.); yafeiChen@zju.edu.cn (Y.C.); 15899229636@163.com (B.L.); 2 School of Energy and Chemical Engineering, Xinjiang Institute of Technology, Aksu 843100, China; xjitshanshan2018@163.com; 3 Ocean College, Zhejiang University, Zhoushan 316021, China

**Keywords:** saline environment, basalt fiber, waterborne epoxy, natural-exposure test, dual-reinforcement network

## Abstract

To improve the durability of concrete in the saline environment of southern Xinjiang, concrete was modified with basalt fiber (BF) and waterborne epoxy (WEP). A one-year outdoor natural-exposure test in Aksu, Xinjiang, systematically investigated the corrosion resistance and multi-scale deterioration behavior and durability of the modified concrete. Compared with C0, the BF2E2 mixture reduced the mass loss rate and flexural strength loss rate by 54.65% and 37.20%, respectively, while increasing the relative dynamic elastic modulus by 14.55%. The modified mixtures also showed decreased total porosity, reduced average pore area, and narrowed crack widths, with significantly attenuated diffraction peaks of corrosion products. Threshold-crossing times obtained by extrapolation indicated that the earliest threshold-crossing time of BF2E2 was extended by 62.14% compared with C0, with the relative dynamic elastic modulus serving as the governing durability criterion. Mechanistic analysis revealed that BF and WEP acted collaboratively through fiber bridging and polymer filling, forming a dual-reinforcement network that suppressed deterioration from the microstructural to the macroscopic scale. These findings provide guidance for the durability-oriented design of concrete structures in saline environments.

## 1. Introduction

Southern Xinjiang, located in the northwestern arid and semi-arid region of China, is exposed to a complex saline environment characterized by high salinity, intense evaporation, dry–wet cycles, and freeze–thaw cycles. Such multifactorial conditions inflict combined degradation effects on concrete structures, including chloride ingress, sulfate crystallization expansion, and alkali-aggregate reaction, resulting in mechanical property deterioration [1,2,3] and reduce service life. In salt-affected soils, chloride ions (Cl^−^) enter concrete by diffusion and capillary absorption, depassivating the protective film on steel reinforcement and initiating electrochemical corrosion. The volumetric expansion of corrosion products causes cracking in the concrete cover [4,5,6]. Meanwhile, sulfate ions (SO_4_^2−^) react with cement hydration products like calcium hydroxide (Ca(OH)_2_) and tricalcium aluminate (C_3_A) to form ettringite (AFt) and gypsum. The expansion of these products promotes the propagation of microcracks [7,8,9]. Additionally, dry–wet cycles and temperature fluctuations in saline regions accelerate pore-water movement and increase the transport of aggressive agents, reinforcing the feedback loop between corrosion and structural damage [10,11,12].

Conventional approaches to enhancing concrete durability, such as reducing the water-to-cement ratio (*w*/*c*) and adding mineral admixtures (e.g., fly ash and silica fume), still fail to meet engineering demands for long-term erosion resistance in natural salt-affected soil environments [13,14,15,16]. Polymer-modified concrete, which uses fiber reinforcement and densification by organic cementitious materials, has emerged as a promising way to improve durability in saline soils. Basalt fiber (BF), an inorganic high-performance fiber, has high tensile strength and excellent corrosion resistance. It suppresses crack initiation and propagation through a bridging effect [17,18,19]. Incorporating BF significantly improves the splitting tensile and flexural strengths of recycled concrete [20]. It also reduces shear failure in recycled coarse- and fine-aggregate concrete [21] and improves the stress state of concrete by transforming eccentric compression into axial compression, indirectly enhancing compressive strength [22]. BF increases the cohesion of concrete mixtures [23] and improves mechanical interlocking and friction at the matrix interface [24]. As an organic modifier, waterborne epoxy (WEP) fills internal pores in concrete [25,26], reduces the transport rate of aggressive agents [27,28], and forms an interpenetrating network (IPN) with cement hydration products, enhancing interfacial bonding strength [29,30,31]. Furthermore, it substantially improves surface hydrophobicity, impermeability, and salt erosion resistance [32,33]. Epoxy-modified hydraulic asphalt concrete also shows significant improvements in water stability, thermal stability, and crack resistance [34].

A single modifier can hardly satisfy concrete’s dual requirements for compactness and crack resistance. Thus, composite modification has become a prevailing research trend in recent years. The hybrid incorporation of basalt fiber with natural bamboo fiber, sisal fiber, or brucite fiber enhances the compactness of the concrete matrix, optimizes the internal microstructure, and retards the coalescence and propagation of microcracks [35,36,37]. The combined addition of basalt fiber and polypropylene fiber refines the pore structure of the concrete matrix and densifies the Interfacial Transition Zone (ITZ) between aggregates and cement paste [38,39]. Furthermore, the synergistic filling effect of basalt fiber and nano-silica densifies the concrete matrix more effectively, suppressing crack formation within the matrix [40]. An appropriate dosage of basalt fiber also improves the mechanical properties and sulfate erosion resistance of fly ash-slag-based pervious concrete [41]. In parallel, graphene oxide–waterborne epoxy composites enhance the corrosion resistance of concrete [42]. The incorporation of epoxy resin and plastic aggregates improves cohesion and anti-spalling capacity [43]. Epoxy-based pervious concrete containing ceramsite exhibits a notable increase in compressive strength [44]. The addition of epoxy resin combined with polypropylene fiber, carbon fiber, or silica sand ameliorates the mechanical performance and interfacial bonding strength of concrete [45,46,47]. Additionally, the hybrid incorporation of nanomaterials with epoxy resin substantially improves the compactness, impermeability, and deformation resistance of concrete [48,49,50]. Although existing studies have validated the beneficial effects of polymer modification on concrete durability, systematic investigations on combined basalt fiber–epoxy modification, long-term performance evolution under natural saline soil exposure, and multi-scale correlation remain scarce. Therefore, comprehensive experimental and mechanistic analyses are urgently warranted.

In this study, concrete specimens were prepared with varying dosages of BF and WEP as independent variables. An outdoor natural burial test was conducted to assess the deterioration resistance of the modified concrete, using mass loss rate (Δ*M*), flexural strength loss rate (Δ*f*), and relative dynamic elastic modulus (*E_r_*) as evaluation indicators. The surface morphology of the specimens was documented throughout the burial period, and microstructural characteristics were systematically compared. This comprehensive methodology facilitated a systematic investigation of the damage evolution process and the multi-scale deterioration mechanisms of concrete under complex environmental conditions. By integrating experimental analysis with mechanistic insights, this study seeks to advance both theoretical understanding and practical applications in BF-WEP modified concrete technology.

## 2. Materials and Methods

### 2.1. Experimental Materials

Conch brand P.O 42.5 ordinary silicate cement, manufactured by Anhui Conch Cement Co., Ltd., Wuhu, Anhui Province, China, was used as the cementitious material. Fine and coarse aggregates were sourced from a sand-and-gravel processing plant in Aksu City. The fine aggregate consisted of natural river sand with particle sizes ranging from 2 to 5 mm, while the coarse aggregate comprised machine-crushed granite stone with nominal particle sizes of 5 to 20 mm. WEP (Model DY-128-50) and a WEP curing agent (Model DY-175) were supplied by Shenyang Dongyan Paint Decoration Co., Ltd., Shenyang, Liaoning Province, China. Silicone-based defoamer (Model LJ-618) was obtained from Beijing Gerueilangjie Technology Co., Ltd., Beijing, China. BF was manufactured by Shanghai Chenqi Chemical Technology Co., Ltd., Shanghai, China. Potable tap water was used for mixing and curing. Technical specifications for these materials are presented in Table 1, Table 2, Table 3 and Table 4.

### 2.2. Specimen Preparation

Concrete with a strength grade of C30 was used in this study. Ten concrete mixtures were prepared with different dosages of BF (0.10%, 0.15%, and 0.20% by volume of concrete) and WEP (2%, 4%, and 6% by mass of cement). The curing agent was added at 30% by mass of WEP to solidify WEP. The silicone-based defoamer was added at 0.5% by mass of WEP to control air entrainment. Prismatic specimens measuring 100 mm × 100 mm × 400 mm were cast for each mixture. For each mixture and each test, three replicate specimens (*n* = 3) were prepared and tested at each exposure time point (0, 3, 6, 9, 12 months). The mix proportions were determined according to Chinese standard JGJ 55-2011 [51], with the specimen naming convention (BFxEy, where x = BF dosage level and y = WEP dosage level) explicitly defined in Table 5. All constituent materials were weighed proportionally.

**Figure 1:**
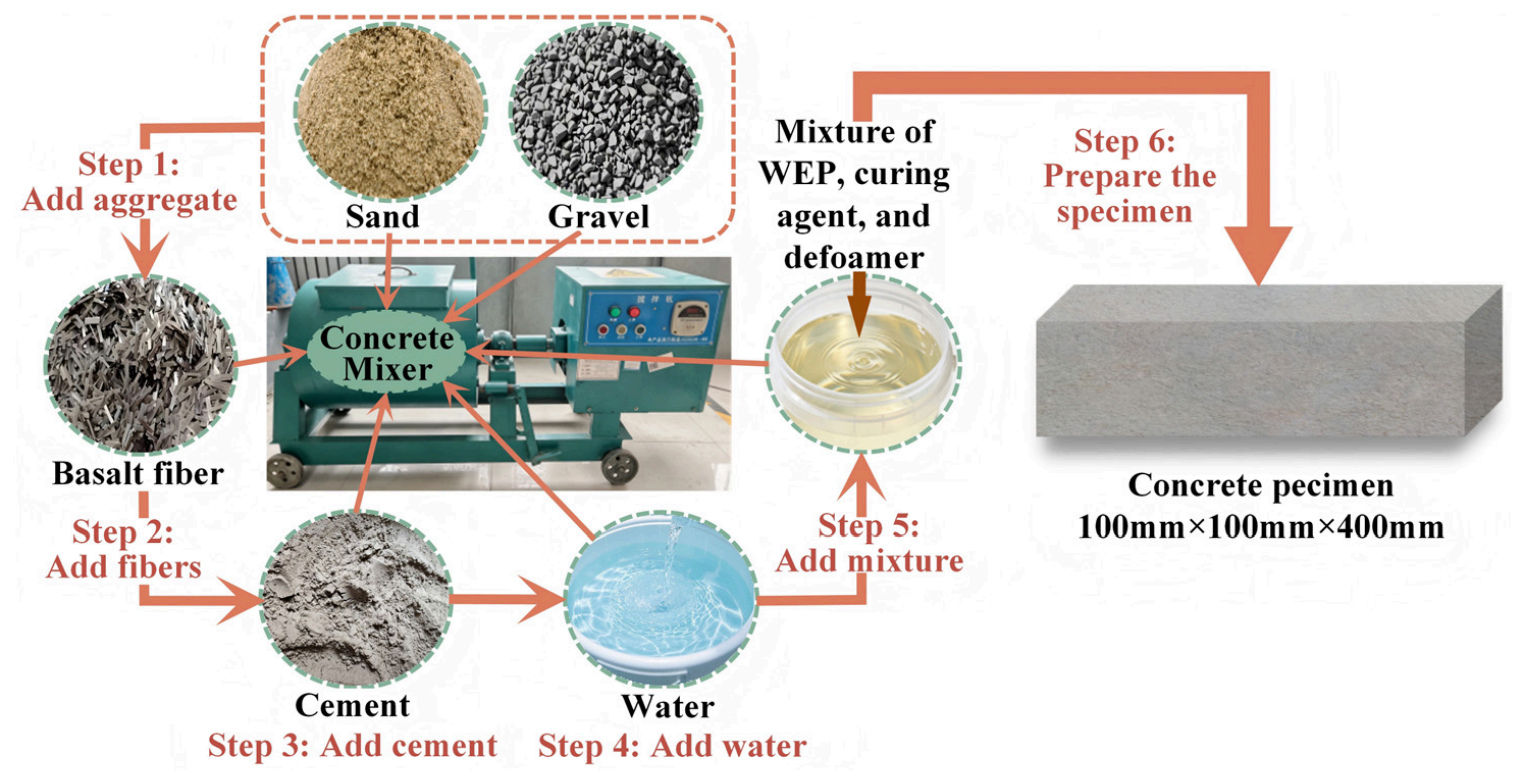
Preparation procedure of concrete specimens.

To enhance fiber dispersion uniformity, the mixing duration was extended. The mixing procedure was as follows. Initially, coarse and fine aggregates were dry-mixed for 30 s. Basalt fibers were then evenly distributed into the mixer, and mixing continued for 60 s to utilize aggregate friction for dispersing fiber bundles into individual filaments, thereby filling the interstitial voids between aggregates. Cement was subsequently added and mixed for 45 s to achieve uniform distribution and to coat the surfaces of aggregates and fibers. Clean water was introduced, and the mixture was stirred for 120 s to ensure that the cement paste fully enveloped the aggregates and fibers. Finally, a pre-blended mixture of epoxy resin, curing agent, and defoamer was added and mixed for 60 to 90 s. The epoxy emulsion must be added at the final stage of mixing, after the concrete constituents have been adequately blended; premature addition would interfere with cement hydration and compromise fiber dispersion. Upon completion of mixing, the fresh concrete was promptly cast into molds and compacted on a vibrating table to achieve full consolidation. The detailed preparation procedure is illustrated in Figure 1. According to GB/T 50082-2009 [52], the specimens were stored in a standard curing chamber for 24 h, then demolded and cured for 28 days before undergoing an outdoor exposure test.

### 2.3. Outdoor Exposure Test

**Figure 2:**
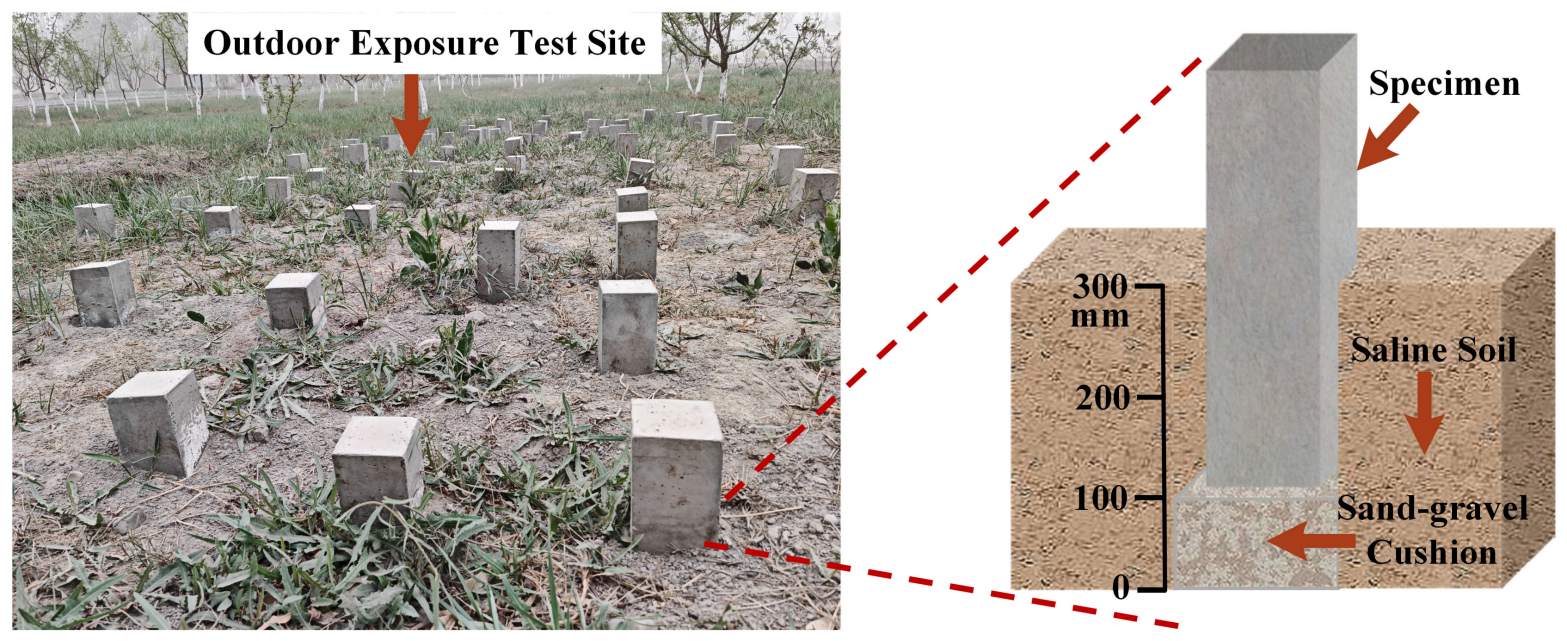
Outdoor exposure test site in Aksu Prefecture.

The test site was located in Aksu Prefecture, southern Xinjiang (41°10′ N, 80°16′ E). This region exhibits a typical continental arid climate, with a mean annual temperature of 10.7 °C and annual precipitation of 57 mm. The soil was classified as saline, with SO_4_^2−^ concentrations ranging from 2.8% to 3.5% and Cl^−^ concentrations from 1.2% to 1.8% in the topsoil. These concentrations satisfy the criteria for “strongly saline soil” as defined in GB/T 50942-2014 [53]. Test specimens were semi-buried at a depth of 200 mm, with the objective of establishing sustained soil contact representative of near-ground/foundation concrete exposure. A 100 mm-thick graded sand-gravel cushion layer was installed beneath the specimens to provide a stable, permeable bedding layer and to approximate the soil-contact/drainage condition encountered by near-ground concrete components, as illustrated in Figure 2. Continuous records of relative humidity, soil moisture, and specimen-scale freeze–thaw events were not acquired during the one-year campaign. Accordingly, the exposure is treated as an integrated natural saline-soil environment, and contributions from individual climatic variables are not separated.

The exposure period lasted one year, with sampling at 0, 3, 6, 9, and 12 months. Specimen surfaces were photographed at each interval to document damage characteristics such as cracking, spalling, and discoloration. Simultaneously, the surface morphology was monitored, and microstructural characteristics were analyzed. The mass, dynamic elastic modulus, and transverse fundamental frequency of the specimens were measured, three indicators (Δ*M*, Δ*f*, *E_r_*) were used to assess the deterioration resistance of the concrete. This comprehensive methodology enabled a systematic investigation of the damage evolution process and the multi-scale deterioration mechanisms of concrete under complex environmental conditions.

### 2.4. Test Methods

#### 2.4.1. Macroscopic Performance Tests

The mass of each specimen was measured using an electronic balance (Model DZC30-1, Shanghai Yousheng Weighing Apparatus Co., Ltd., Shanghai, China; accuracy: 0.1 g). Before weighing, specimens were carefully excavated, loose soil particles were removed with a soft nylon brush, and adhered salts and soil fines were rinsed with deionized water for 30 s. Specimens were then oven-dried at 60 ± 2 °C for 24 h to constant mass (mass change < 0.1 g over 6 h), cooled to room temperature in a desiccator for 2 h, and weighed using an electronic balance. The same cleaning, drying, and conditioning protocol was applied at all time points. The mass loss rate was then calculated using Equation (1).

Δ*M* = 100% × (*M*_0_ − *M*_d_)/*M*_0_ (1)

where Δ*M* is the mass loss rate (%), *M*_0_ is the initial mass (kg), and *M*_d_ is the mass of the specimen after d months of exposure to saline soil (kg).

Flexural strength was measured in accordance with GB/T 50081-2019 [54] using a universal testing machine (Model WDW-100, Shanghai Hualong Test Instrument Co., Ltd., Shanghai, China) under three-point loading. Testing was conducted at a span length of 300 mm and a loading rate of 0.08 MPa/s. The flexural strength loss rate was calculated using Equation (2).

Δ*f* = 100% × (*f*_c_ − *f*_d_)/*f*_c_ (2)

where Δ*f* is the flexural strength loss rate (%), *f*_c_ is the average flexural strength of control specimens cured under standard conditions for the same age (MPa, accuracy: 0.01 MPa), and *f*_d_ is the average flexural strength of specimens after d months of exposure to saline soil (MPa, accuracy: 0.01 MPa).

The transverse fundamental frequency was measured using a dynamic elastic modulus tester (Model DT-16, Tianjin Huatong Test Instrument Factory, Tianjin, China). In accordance with GB/T 50082-2024 [55], the relative dynamic elastic modulus was calculated using Equation (3).

*E_r_* = 100% × (*f*_n_/*f*_0_)^2^ (3)

where *E_r_* is the relative dynamic elastic modulus (%), *f*_0_ is the initial transverse fundamental frequency (Hz), and *f*_n_ is the transverse fundamental frequency of the specimen after n months of exposure to saline soil (Hz).

#### 2.4.2. Microstructural Characterization

Scanning electron microscopy (SEM) test: The microstructural morphology of the ITZ and corrosion products was characterized using a field-emission scanning electron microscope (Sigma 300, Carl Zeiss AG, Oberkochen, Germany) operated at 15 kV. Before imaging, specimens were dried and sputter-coated with a thin gold film to improve surface conductivity.

X-ray diffraction (XRD) test: The phase composition of corrosion products was determined by X-ray powder diffraction using a fixed-target diffractometer (SmartLab SE, Rigaku Corporation, Akishima, Tokyo, Japan). Measurements were conducted over a 2θ range of 5° to 90° at a scan rate of 2°/min.

## 3. Results and Discussion

### 3.1. Appearance Change Characteristics

**Figure 3:**
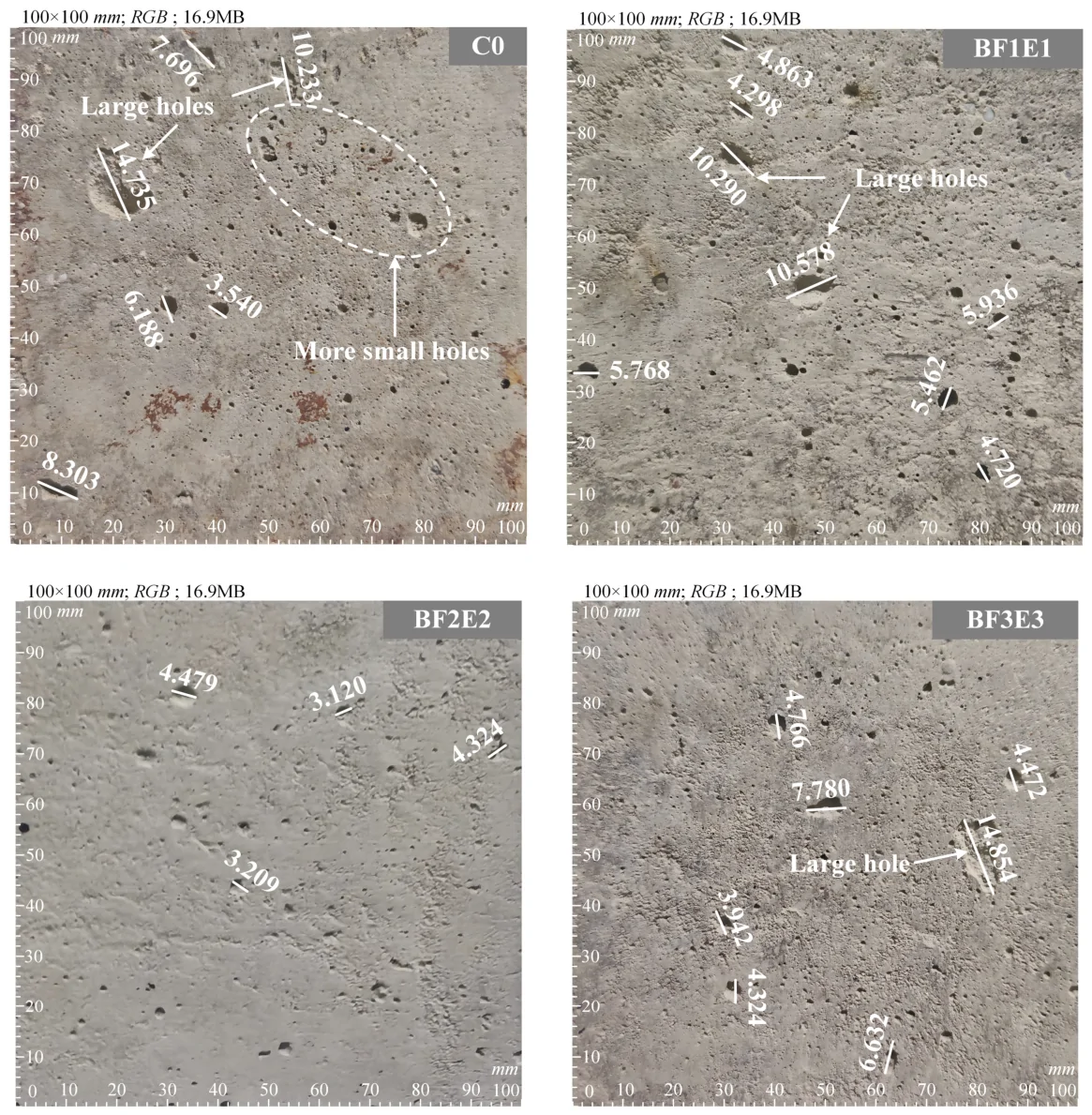
Appearance of specimens after 12 months of exposure.

The bottom surface pore dimensions of the specimen embedded in the soil were quantified using ImageJ software (version 1.54g, National Institutes of Health, Bethesda, MD, USA). Figure 3 shows appearance changes of concrete specimens (C0, BF1E1, BF2E2, and BF3E3) after 12 months of exposure to saline soil.

The C0 specimen showed pronounced spalling and cracking across its surface, with pores up to 14.735 mm in diameter. These pores merged to form extensive defects. Increasing BF and WEP dosages markedly improved the surface integrity of the modified specimens. For BF2E2, only slight spalling was observed, the number of pores was significantly reduced, and the maximum pore size decreased to 4.479 mm. However, further increases in BF and WEP dosages led to greater damage, with a noticeable increase in both the number and size of surface pores.

### 3.2. Microstructural Evolution Characteristics

#### 3.2.1. Phase Analysis of Corrosion Products

**Figure 4:**
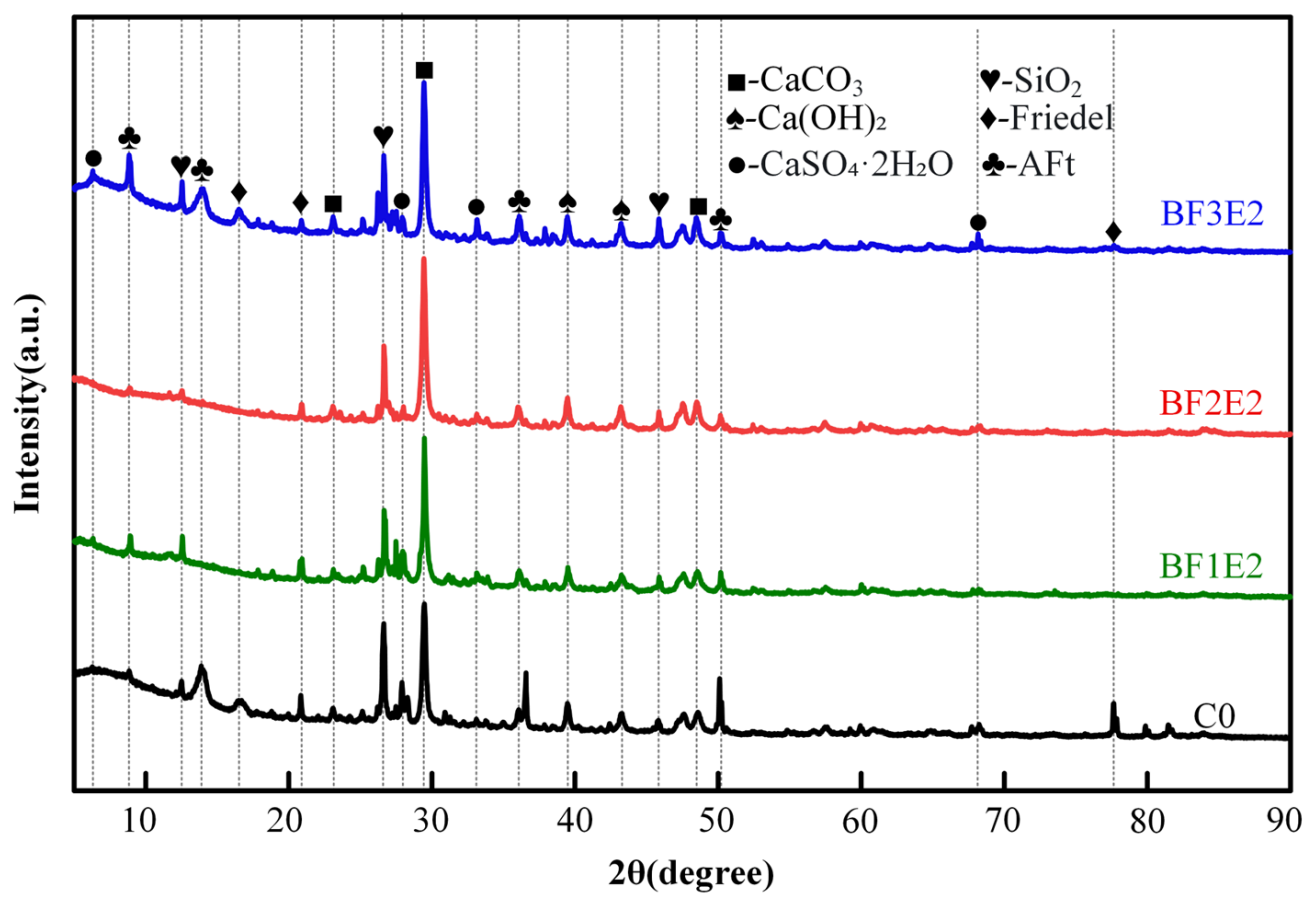
XRD patterns of specimens after 12 months of exposure.

Figure 4 displays the XRD patterns of C0, BF1E2, BF2E2, and BF3E2 after 12 months of exposure. The XRD analysis identified characteristic diffraction peaks corresponding to ettringite (AFt, 3CaO·Al_2_O_3_·3CaSO_4_·32H_2_O, 2θ = 8.8°, 14.0°, 36.1°, 50.1°), gypsum (CaSO_4_·2H_2_O, 2θ = 6.3°, 27.5°, 33.2°, 68.1°), and Friedel’s salt (3CaO·Al_2_O_3_·CaCl_2_·10H_2_O, 2θ = 16.5°, 20.9°, 77.6°) in all tested specimens.

The C0 specimen exhibited strong diffraction peaks for ettringite, gypsum, and Friedel’s salt. In contrast, the BF2E2 specimen demonstrated significantly lower peak intensities for these phases. This reduction can be attributed to the pore-filling effect of WEP, which decreased porosity and restricted the penetration of aggressive ions. Additionally, BF contributed to the prevention of crack initiation and propagation, thereby reducing pathways for aggressive agents.

#### 3.2.2. SEM Comparison

**Figure 5:**
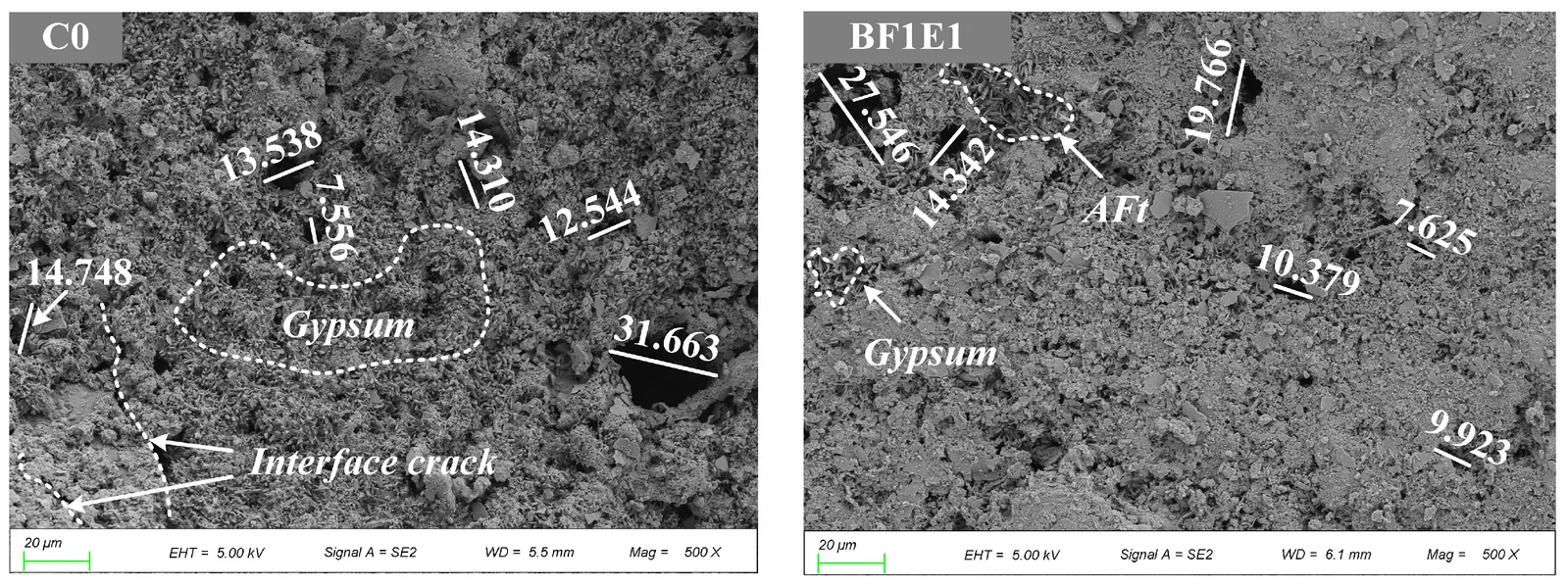

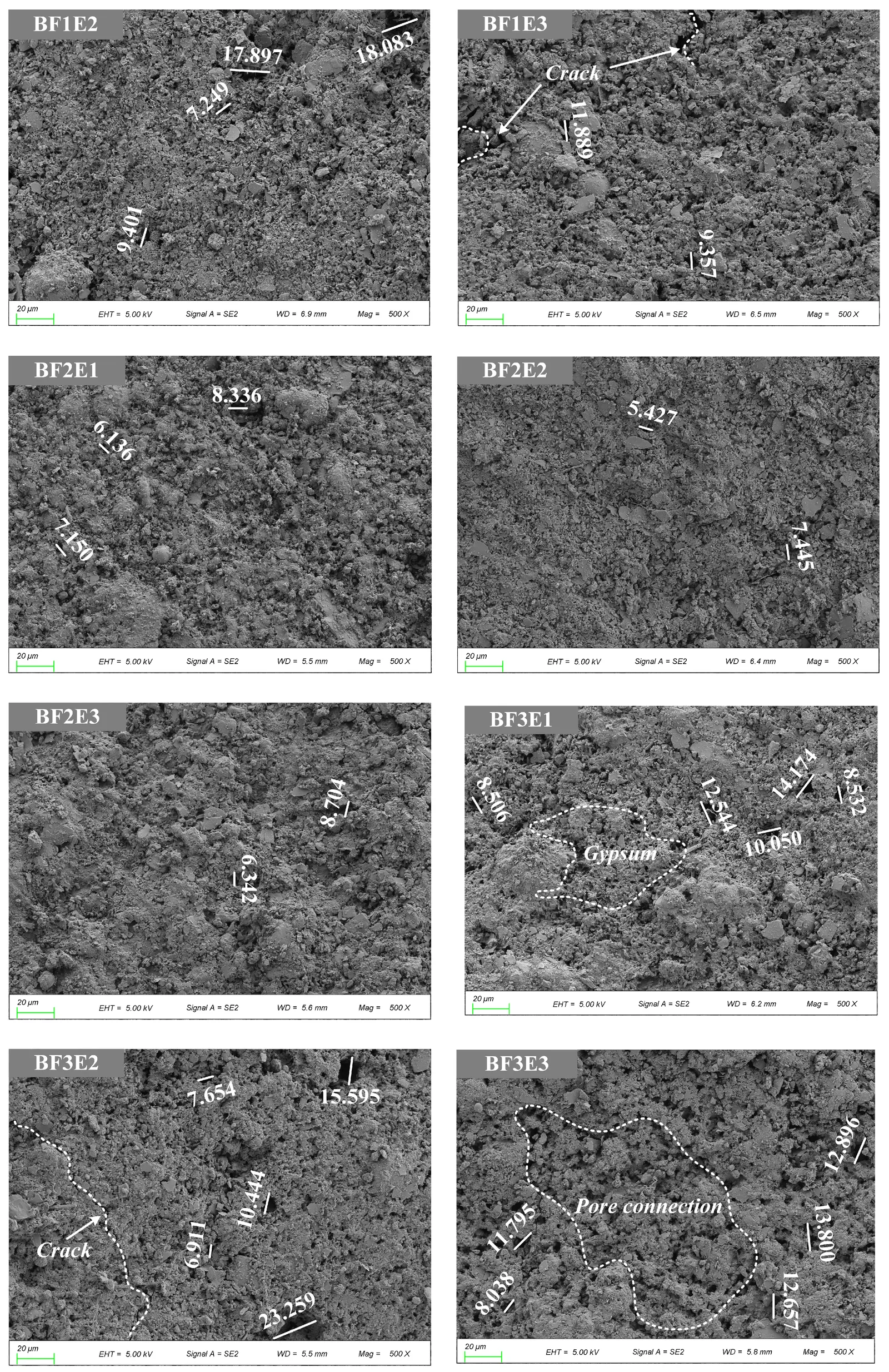
SEM micrographs of specimens after 12 months of exposure.

Figure 5 presents a comparative analysis of the SEM microstructural morphology of the specimens after 12 months of exposure.

SEM observations of the C0 revealed a loose and porous cement paste matrix with numerous cracks and voids. Crack widths were large (approximately 7–32 μm), and the cracks formed an interconnected network. Gaps appeared at the aggregate-cement paste interface, indicating poor bonding. Substantial corrosion products (AFt, gypsum, etc.) formed within pores and cracks, further compromising the concrete’s microstructure. In contrast, the BF2E2 showed a dense cement paste matrix with fewer cracks and pores. Crack widths were smaller (˂7 μm), and cracks were mostly isolated and closed, not forming a network. The aggregate–cement paste interface was tightly bonded, with fewer interfacial gaps.

#### 3.2.3. Evolution of Pore Structure

**Figure 6:**
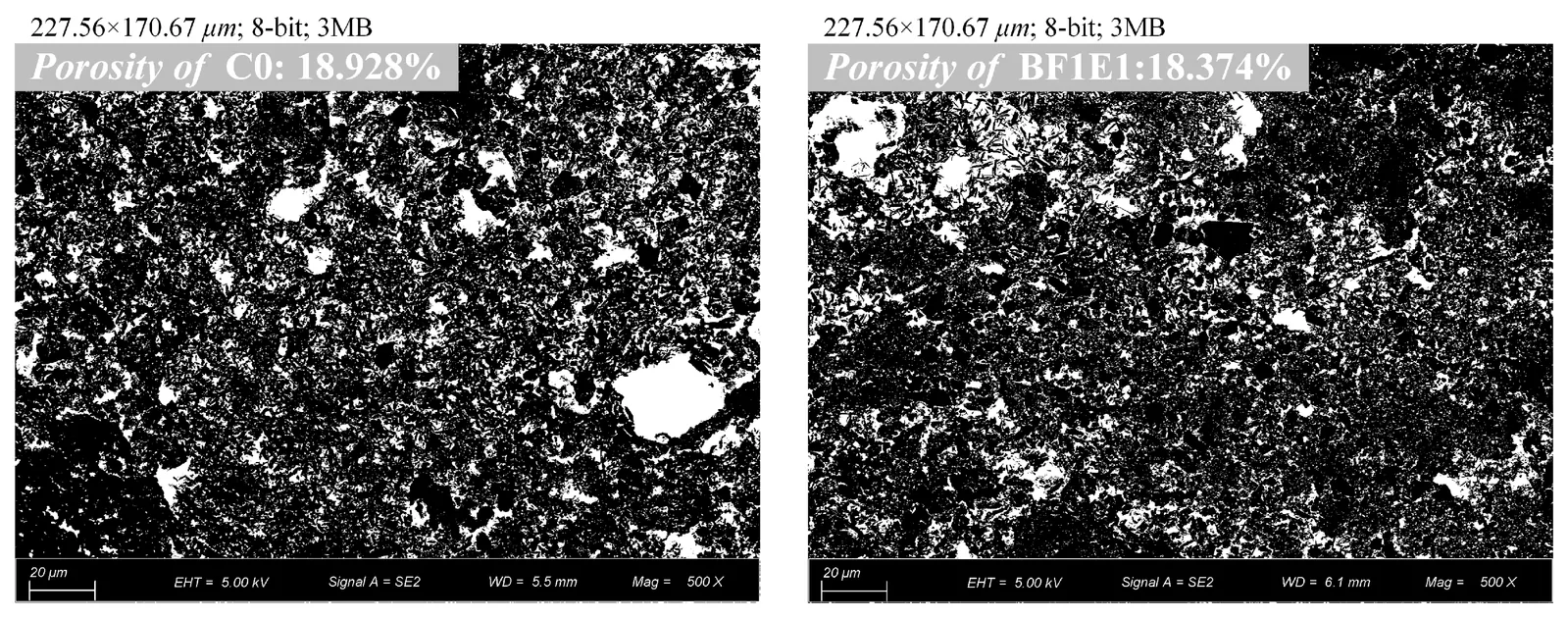

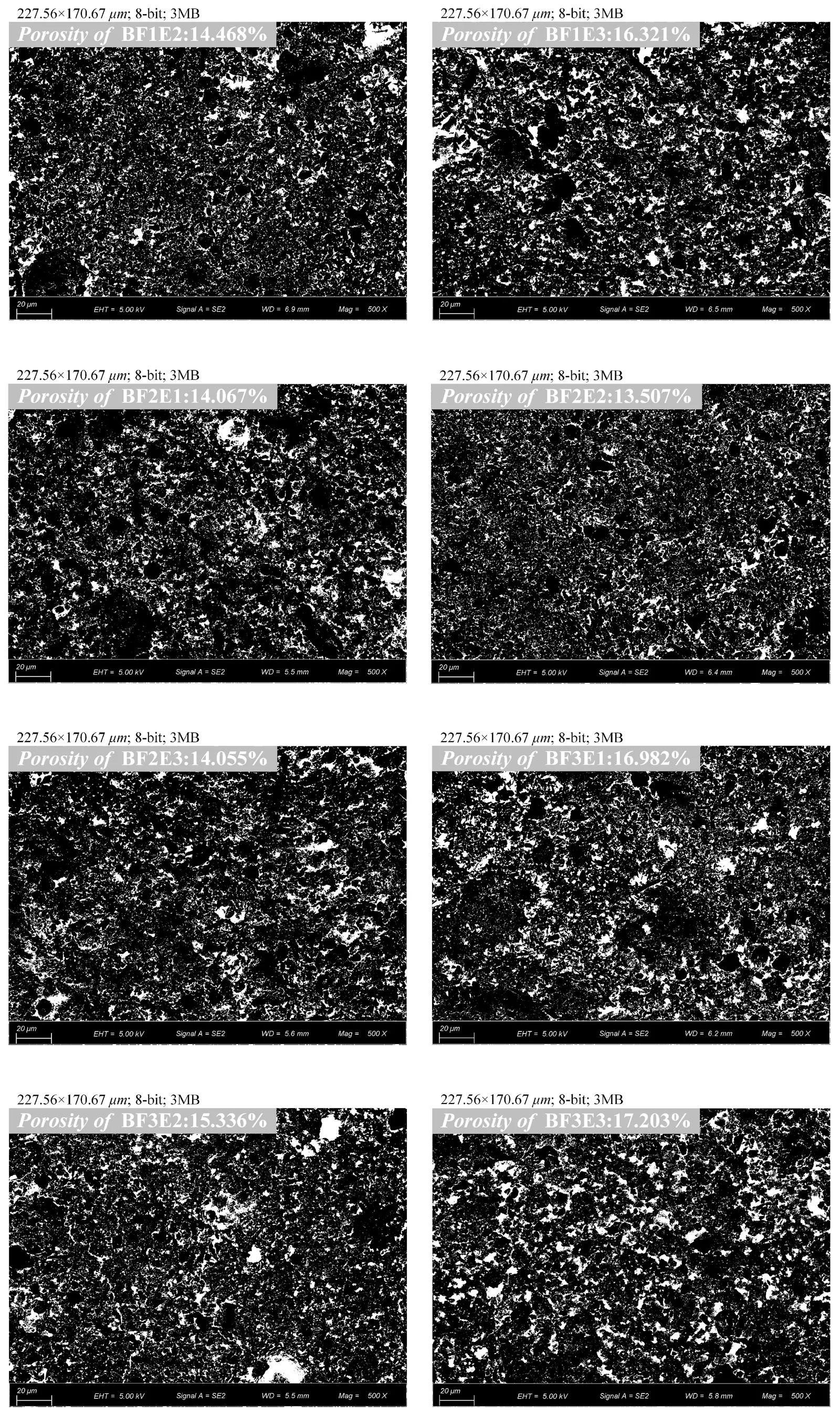
Image-based two-dimensional pore feature of specimens after 12 months of exposure.

SEM images for pore analysis were acquired at 500× magnification, with a scale bar of 20 μm. Three images were captured for each specimen, and one representative image was selected for comparative analysis. The pore size distribution of the specimens was quantified using ImageJ (v1.54g). The image type was an 8-bit grayscale image, and the threshold was set to 55 for all images. Figure 6 presents the image-based two-dimensional pore-feature of specimens after 12 months of exposure, detailed pore structure parameters are summarized in Table 6.

The results demonstrate that the total porosity of BF2E2 decreased by 28.64% and the average pore area decreased by 36.67% compared to C0. This enhancement in pore structure is primarily attributed to the combined effects of the pore-filling action of WEP and the bridging action of BF [56]: at appropriate WEP dosages, a polymer film forms within the concrete matrix, filling capillary pores, particularly those with large diameters; at suitable BF dosages, the bridging action inhibits the initiation and propagation of microcracks, further reducing the formation of crack-induced pores. When the BF dosage exceeds 0.15% or the WEP dosage exceeds 4%, excessive BF tends to agglomerate [21,22], and surplus WEP coats the aggregate surfaces [4,34,48]. Both phenomena result in a slight increase in porosity, which explains the higher total porosity of BF3E3 compared to BF2E2.

### 3.3. Evolution of ΔM

**Figure 7:**
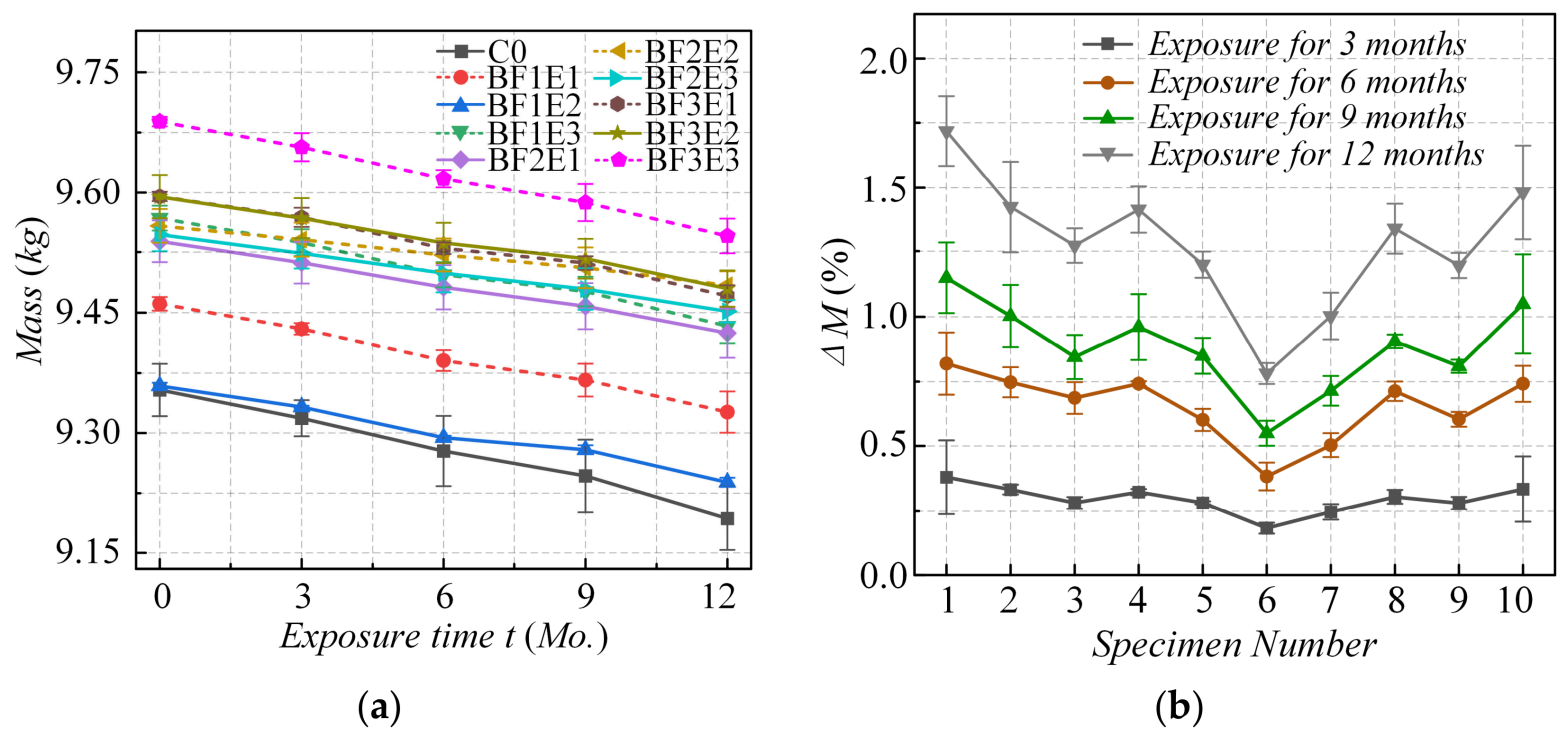
The variations in mass and Δ*M* of all specimen groups over exposure time: (**a**) Variation of mass. (**b**) Variation of ΔM.

Figure 7 show the variations in mass and Δ*M* of all specimen groups over exposure time. As exposure duration increased, Δ*M* increased across all specimens. However, the growth rate of the modified groups was much lower than that of the control group. After 12 months, Δ*M* of C0 reached 1.720%, while BF2E2 was only 0.780%, corresponding to a reduction of 54.65% compared to C0. The Δ*M* of all specimen groups at 12 months ranked as follows: C0 (1.720%) > BF3E3 (1.481%) > BF1E1 (1.427%) > BF1E3 (1.416%) > BF3E1 (1.342%) > BF1E2 (1.277%) > BF2E1 (1.202%) > BF3E2 (1.199%) > BF2E3 (1.003%) > BF2E2 (0.780%).

The results indicate that the combined use of BF and WEP produces a significant combined effect in reducing the Δ*M*. At appropriate WEP dosages, the internal pores of the concrete are filled, resulting in a dense three-dimensional network structure that inhibits the ingress of aggressive agents [4,34]. Simultaneously, BF impedes the propagation of surface damage by bridging cracks and dispersing stress [21]. However, when the WEP dosage exceeds 4% or the BF dosage exceeds 0.15%, the mass loss rate increases. This increase is attributed to excessive WEP coating portions of the aggregates, which impedes hydration and reduces compactness. Additionally, surplus BF forms localized clusters, leading to non-uniform distribution and reduced reinforcement efficiency.

### 3.4. Deterioration Law of Δf

**Figure 8:**
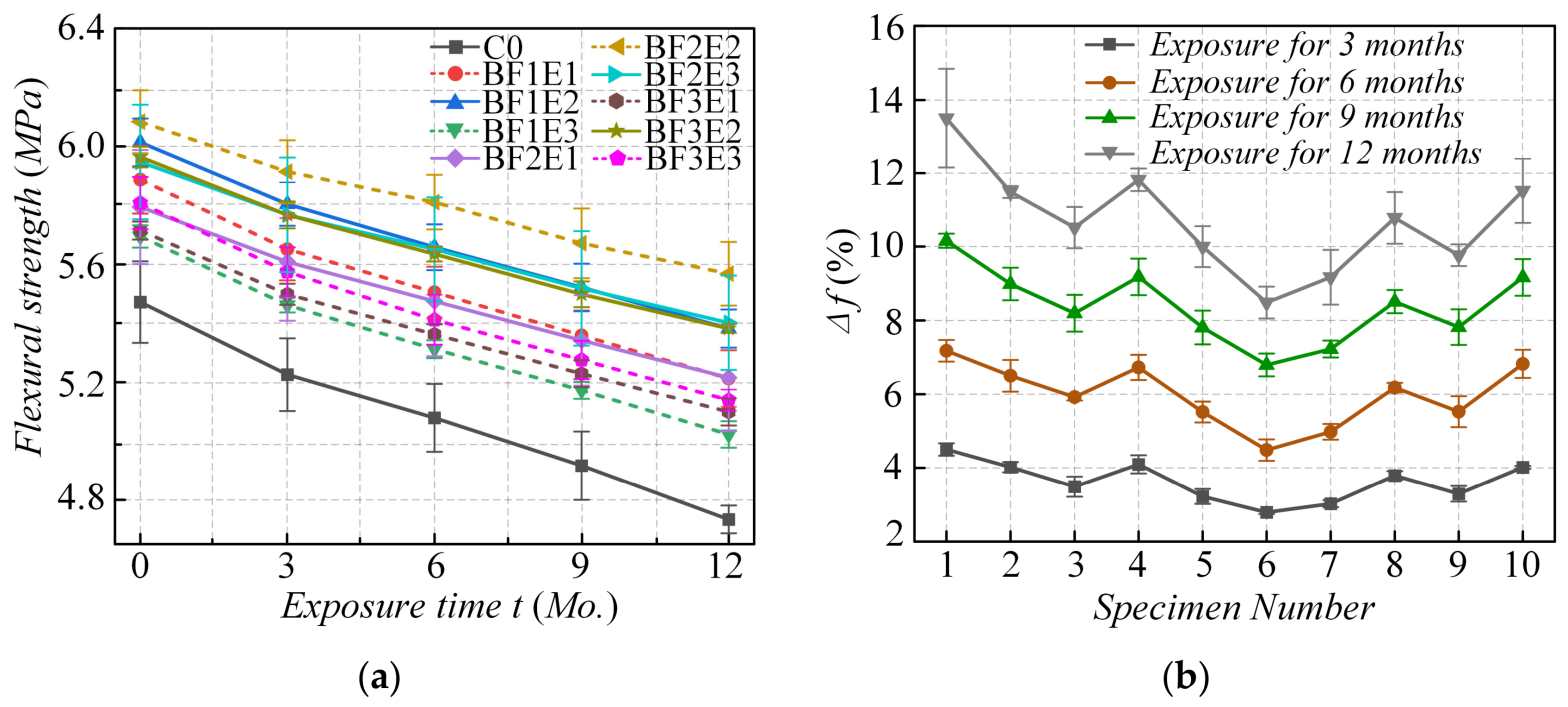
The variation curves of flexural strength and Δ*f* for all specimen groups over exposure time: (**a**) Variation of flexural strength. (**b**) Variation of Δ*f*.

Figure 8 show the variation curves of flexural strength and Δ*f* for all specimen groups over exposure time. After 12 months, Δ*f* of C0 reached 13.52%, while BF2E2 was 8.49%, a reduction of 37.20%. The Δ*f* values at 12 months ranked as follows: C0 (13.52%) > BF1E3 (11.82%) > BF3E3 (11.53%) > BF1E1 (11.49%) > BF3E1 (10.79%) > BF1E2 (10.53%) > BF2E1 (10.01%) > BF3E2 (9.78%) > BF2E3 (9.19%) > BF2E2 (8.49%).

The findings demonstrate that an appropriate dosage of BF and WEP in concrete establishes a combined enhancement mechanism described as “fiber bridging–polymer filling” [56]. This mechanism improves flexural toughness and interfacial bonding strength, thereby effectively delaying the deterioration of flexural strength in saline environments.

### 3.5. Evolution Trend of E_r_

**Figure 9:**
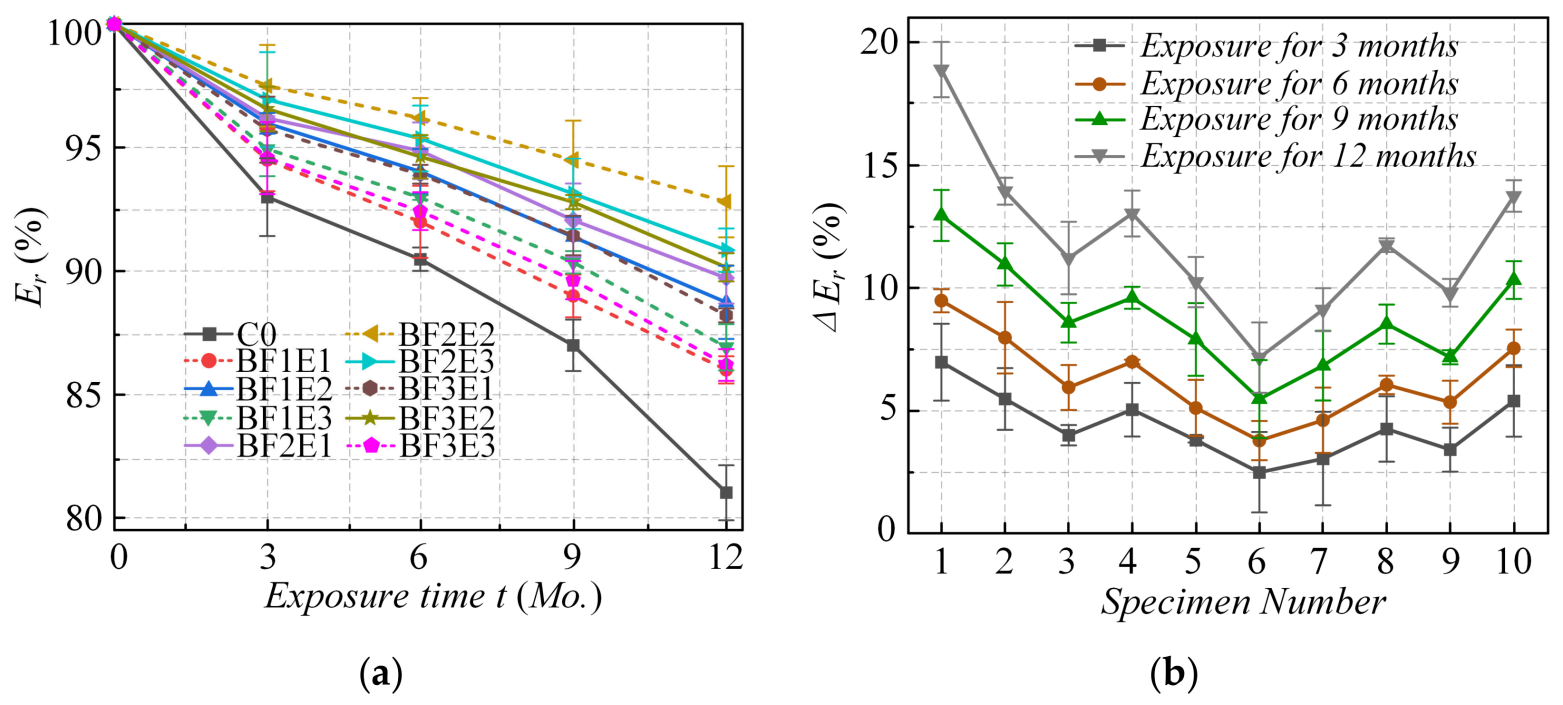
The variation curves of *E_r_* and dynamic elastic modulus loss rate for all specimen groups over time: (**a**) Variation of *E_r_*. (**b**) Variation of dynamic elastic modulus loss rate.

Figure 9 show the variation curves of Er and dynamic elastic modulus loss rate for all specimen groups over time. After 12 months of exposure, *E_r_* of C0 decreased to 81.01%, while BF2E2 remained at 92.80%, an increase of 14.55% compared to C0. *E_r_* values of all specimen groups at 12 months ranked in descending order as follows: BF2E2 (92.80%) > BF2E3 (90.85%) > BF3E2 (90.15%) > BF2E1 (89.72%) > BF1E2 (88.73%) > BF3E1 (88.21%) > BF1E3 (86.92%) > BF3E3 (86.20%) > BF1E1 (86.00%) > C0 (81.01%).

The *E_r_* reflects the cumulative degree of internal damage within concrete. In C0 specimens exposed to saline soil environments, SO_4_^2−^ and Cl^−^ penetrated the concrete and reacted with hydration products to form expansive compounds. This reaction resulted in the continuous propagation of internal microcracks and a progressive decline in *E_r_*. In contrast, in modified concrete, BF filaments spanning microcracks enabled interfacial bonding with the concrete matrix, which facilitated stress transfer and limited further crack extension [21]. Concurrently, WEP formed a polymer film within the matrix, filling capillary pores and obstructing transport pathways for aggressive agents [34], thereby reducing the accumulation of internal damage. The BF2E2 group maintained a relatively high *E_r_* after 12 months of exposure, indicating effective preservation of internal structural integrity.

### 3.6. Correlation Analysis Between Δf and E_r_

**Figure 10:**
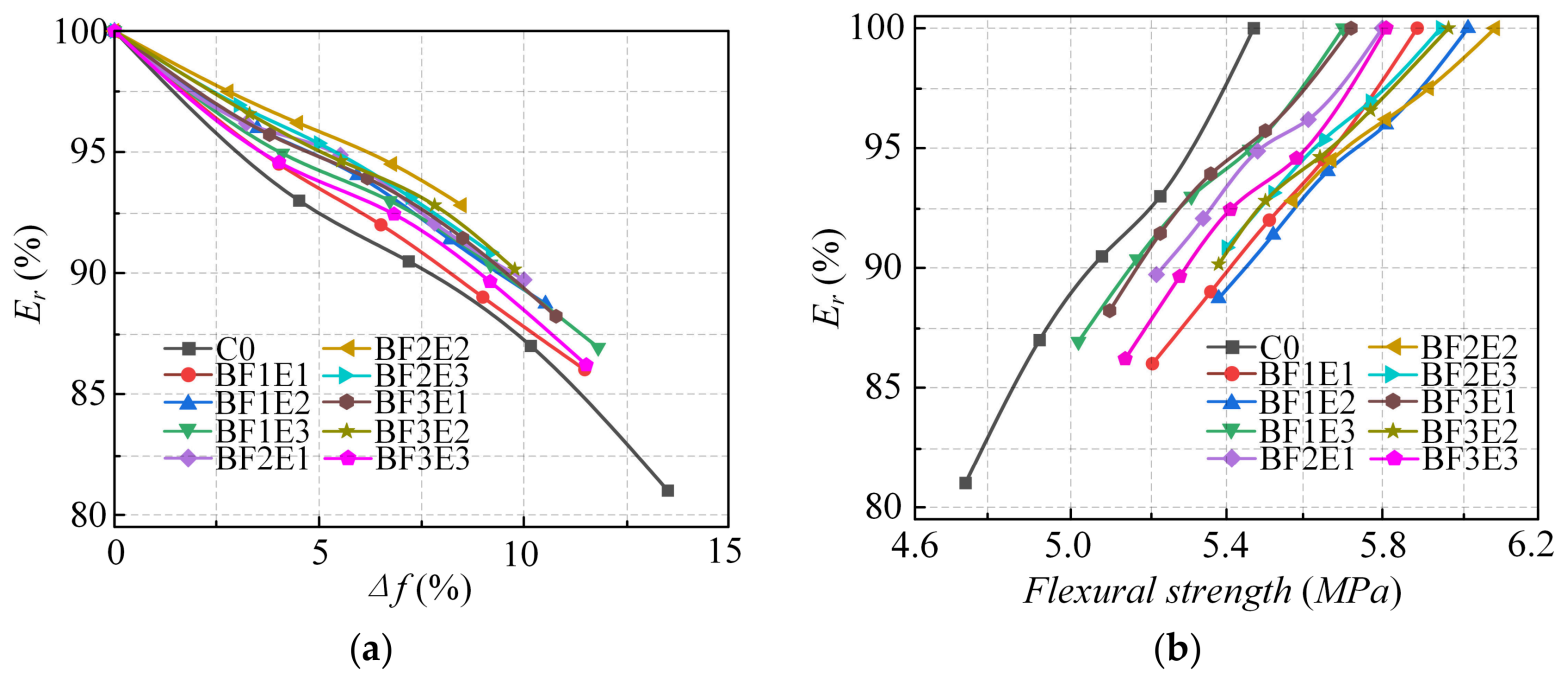
The relationship curves between Δ*f*, flexural strength, and *E_r_*: (**a**) Relationship curves between Δ*f* and *E_r_*. (**b**) Relationship curves between flexural strength and *E_r_*.

Figure 10 presents the relationship curves between Δ*f*, flexural strength, and *E_r_*.

The figures illustrate that Δ*f* exhibits a significant negative correlation with *E_r_*; specifically, as Er decreases, Δ*f* progressively increases. Based on experimental data from 10 specimen types after 12 months of exposure, a polynomial regression analysis was conducted using MATLAB R2026a (The MathWorks, Inc., Natick, MA, USA) to establish the correlation between these parameters, yielding Equation (4).

Δ*f* = 7.648 × 10^−4^*E_r_*^3^ − 2.304 × 10^−1^*E_r_*^2^ + 2.214 × 10*E_r_* − 6.745 × 10^2^ (*R*^2^ = 0.9654) (4)

**Figure 11:**
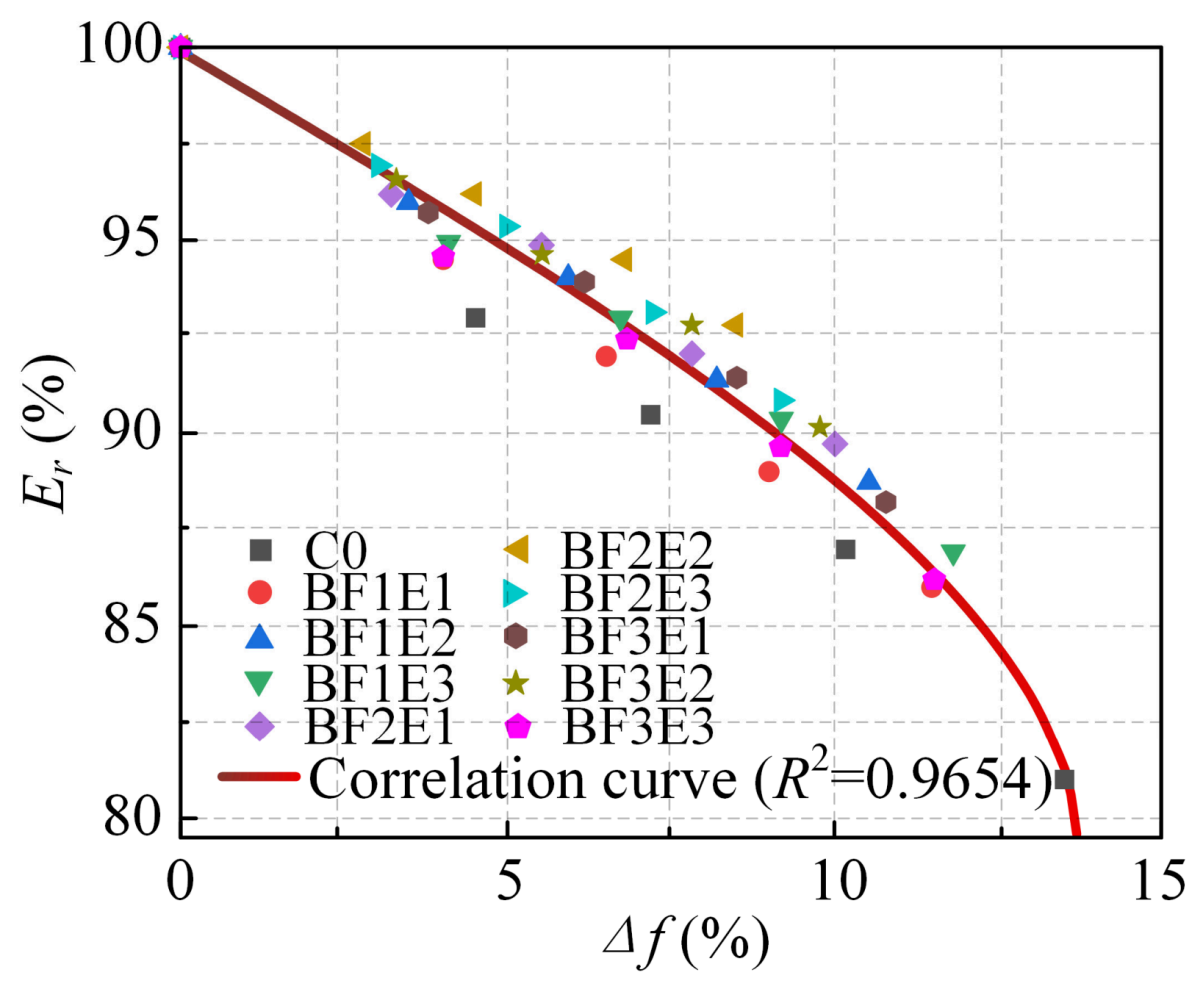
Correlation curve between Δ*f* and *E_r_*.

The coefficient of determination (*R*^2^) for this correlation equation is 0.9654, indicating a strong relationship between the two parameters. The correlation curve between Δ*f* and *E_r_* is shown in Figure 11. This relationship indicates that internal damage in concrete, as indicated by *E_r_*, is closely associated with flexural strength deterioration. For example, in the C0 group, after 12 months of exposure, *E_r_* was 81.01%. Substituting this value into the correlation equation yields a predicted Δ*f* of 13.63%, which closely matches the actual value of 13.52%.

The significance of this correlation model is that *E_r_* reflects accumulated damage from internal microcracks and pores, while Δ*f* reflects deterioration in of the material’s resistance to flexural loading. The strong correlation between these parameters indicates that, under saline soil erosion conditions, internal structural damage is the primary cause of mechanical property degradation in concrete. This model offers a theoretical basis for rapid assessment of concrete durability in saline environments. Specifically, the extent of flexural strength damage can be indirectly estimated through non-destructive dynamic elastic modulus testing. The correlation model is empirical and based on the 10 tested mixtures; its generalizability to other concrete compositions, exposure conditions, or longer durations requires further investigation.

## 4. Exploratory Damage-Curve Fitting and Threshold-Crossing Extrapolation

To quantitatively describe the damage evolution of modified concrete in a saline soil environment, cubic polynomial damage evolution equations were established for Δ*M*, Δ*f*, and *E_r_* based on the measured data obtained from ten mixtures at five exposure time points (0, 3, 6, 9, and 12 months) through the one-year outdoor natural exposure test. These equations are empirical interpolating/descriptive functions over the measured interval and are not derived from a physical deterioration law. Extrapolation beyond 12 months can produce model-form uncertainty. Accordingly, the extrapolated threshold-crossing times reported below are retained only as exploratory scenario-based indicators for relative comparison.

### 4.1. Damage Evolution Model for ΔM

A cubic polynomial damage evolution Equation (5) was established by fitting the relationship between Δ*M* and exposure time for each specimen group using MATLAB R2026a.

Δ*M*(*t*) = *a*_1_*t*^3^ + *b*_1_*t*^2^ + *c*_1_*t* + *d*_1_ (5)

where *t* is the exposure time (months); *a*_1_, *b*_1_, *c*_1_, and *d*_1_ are fitting parameters.

Table 7 summarizes the fitted equations and *R*^2^ for the Δ*M* vs. *t* for all specimen groups.

As shown in Table 7, *R*^2^ for the cubic polynomial models of mass loss rate for all specimens exceeded 0.9880, indicating excellent fit. For exploratory comparison, Δ*M* = 5% was adopted as an analytical threshold. Although mass-loss criteria are used in durability standards such as GB/T 50082-2024 [55], transferring a specific threshold from standardized durability testing to one-year natural saline-soil exposure is a modeling assumption rather than a direct serviceability requirement for the present field condition. The calculated crossing times are therefore reported as extrapolated indicators only.

**Figure 12:**
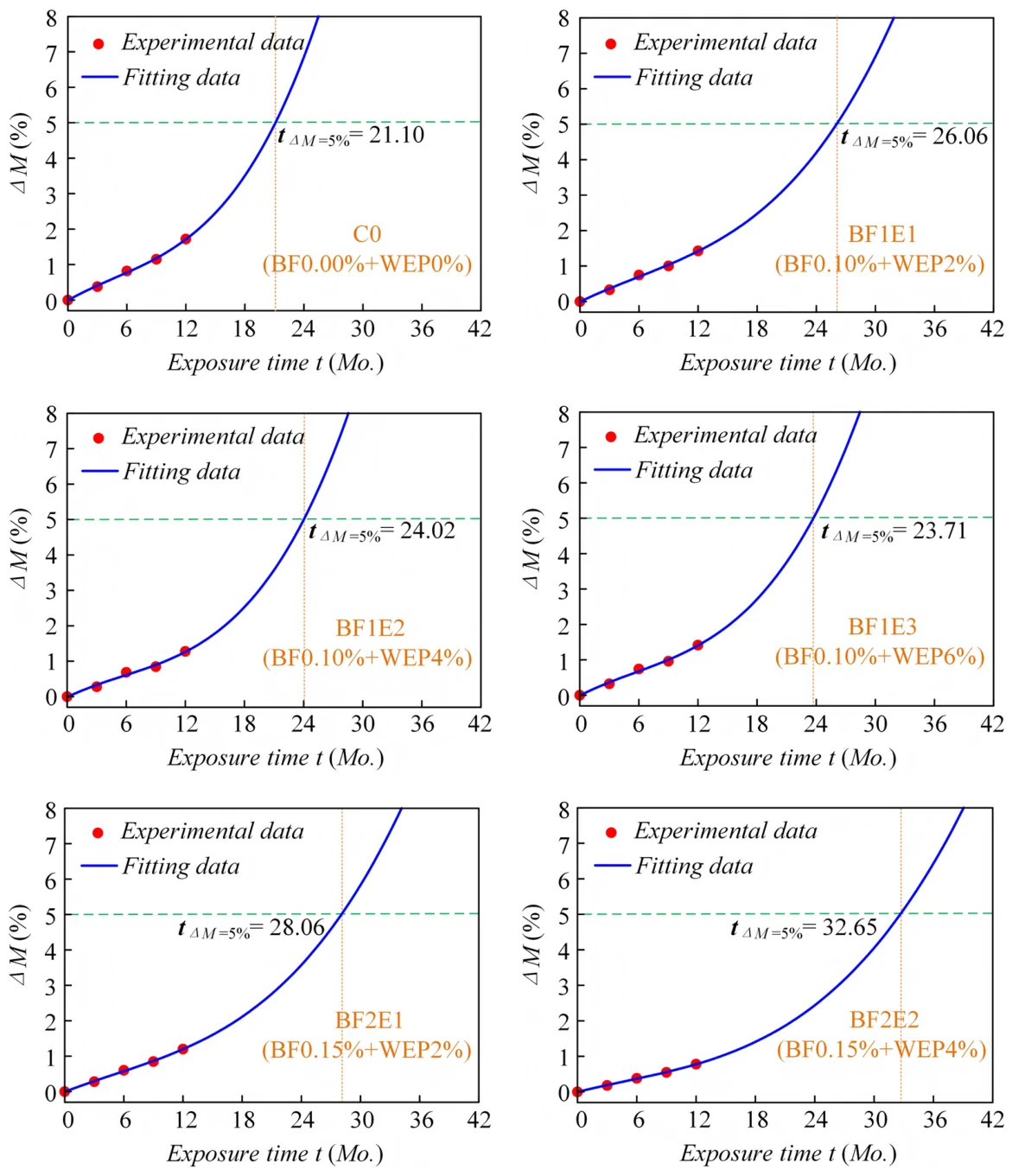

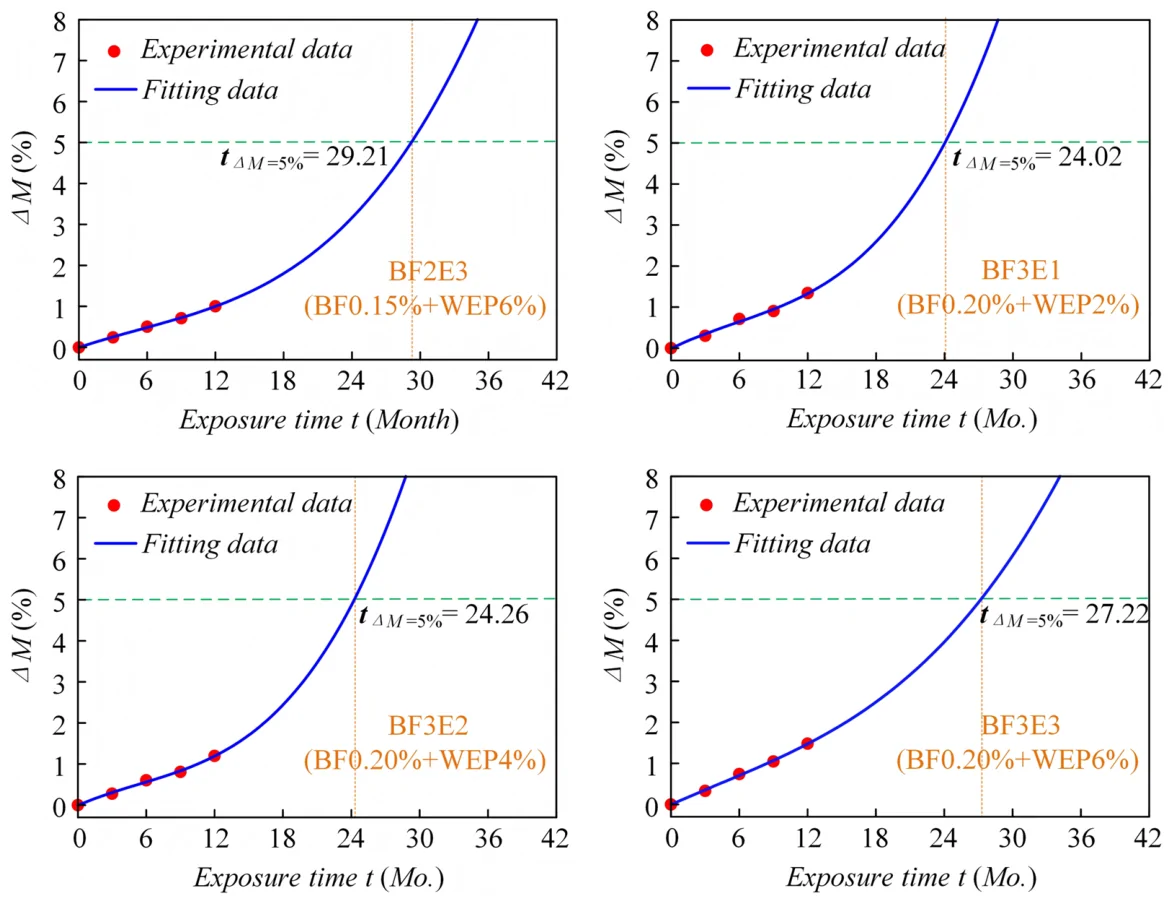
Cubic fits of Δ*M* over the measured interval and exploratory extrapolation to the adopted threshold.

Substituting Δ*M* = 5% into the fitted equation, the corresponding exposure time (*t*) was calculated as the earliest threshold-crossing time (*ETT*) of the concrete. The fitted curves for Δ*M* and the *ETT* for all specimens are shown in Figure 12.

### 4.2. Damage Evolution Model for Δf

A cubic polynomial damage evolution Equation (6) was established by fitting the relationship between Δ*f* and exposure time for each specimen group using MATLAB R2026a.

Δ*f*(*t*) = *a*_2_*t*^3^ + *b*_2_*t*^2^ + *c*_2_*t* + *d*_2_ (6)

where *t* is the exposure time (months); *a*_2_, *b*_2_, *c*_2_, and *d*_2_ are fitting parameters.

Table 8 summarizes the fitted equations and *R*^2^ for Δ*f* vs. *t* for all specimen groups.

As shown in Table 8, the *R*^2^ for the cubic polynomial models of Δ*f* for all specimens exceeded 0.9970, indicating an exceptional good fit. Based on engineering practice, Δ*f* = 30% was used as a selected severe-loss scenario for comparative extrapolation. Because the criterion is assumption-dependent and the polynomial is extrapolated beyond the measured interval, the resulting crossing times are illustrative rather than validated service-life values.

**Figure 13:**
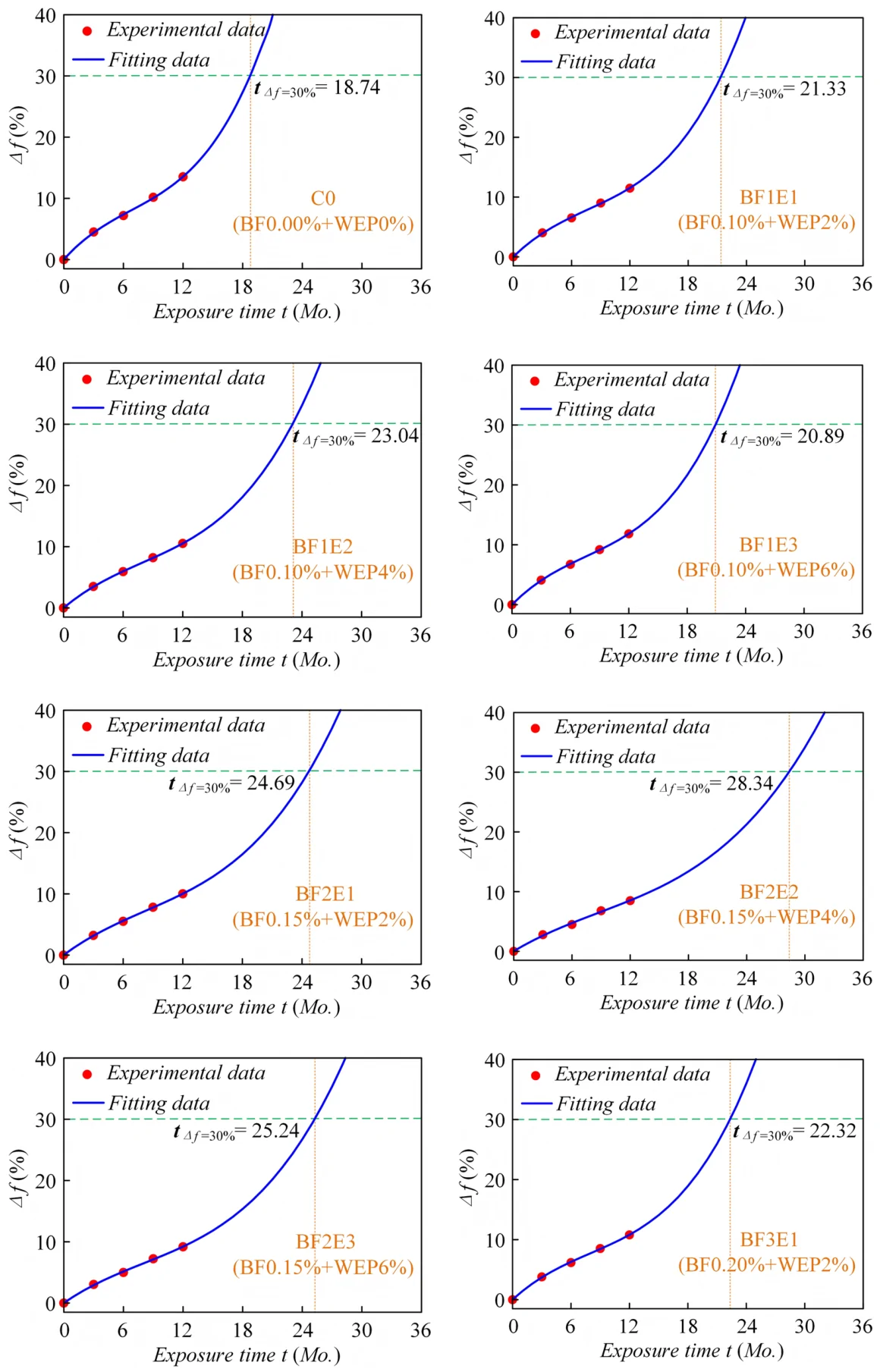

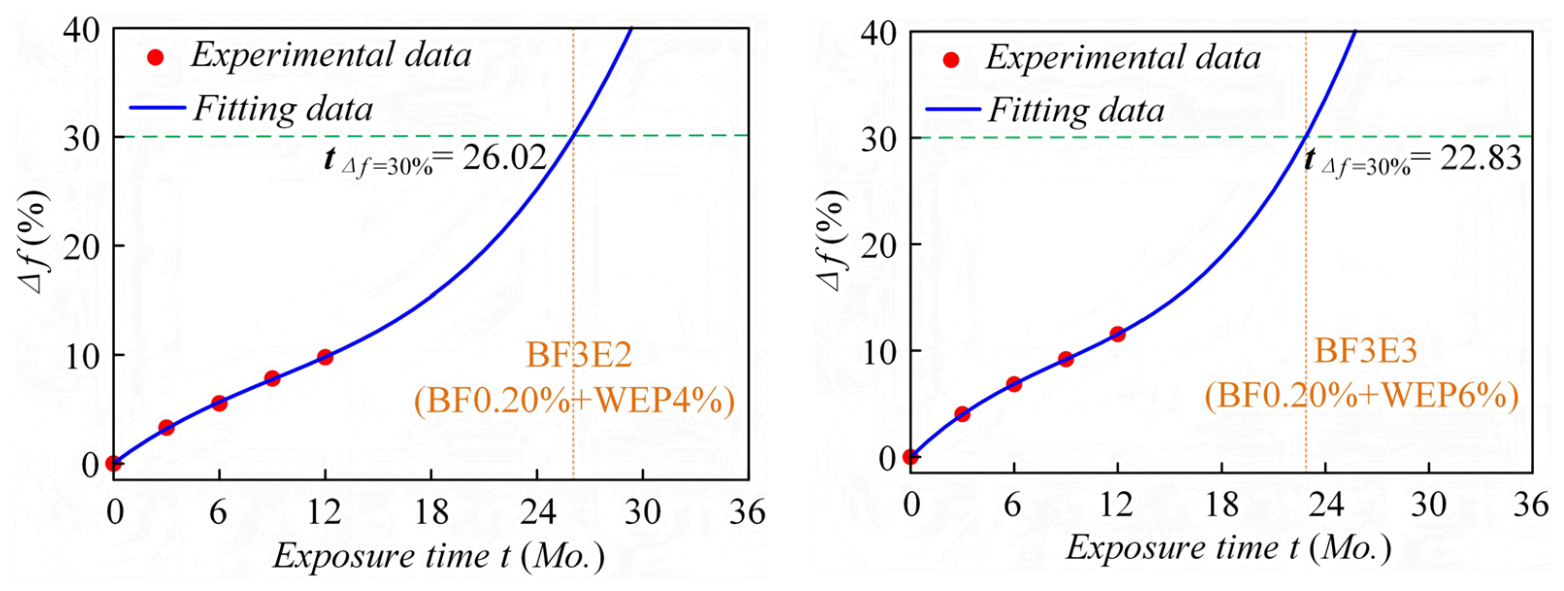
Cubic fits of Δ*f* over the measured interval and exploratory extrapolation to the adopted threshold.

Substituting Δ*f* = 30% into the fitted equation, the corresponding exposure time (*t*) was calculated as the *ETT* of the concrete. The fitted curves for Δ*f* and the *ETT* for all specimens are presented in Figure 13.

### 4.3. Damage Evolution Model for E_r_

A cubic polynomial damage evolution Equation (7) was established by fitting the relationship between *E_r_* and exposure time for each specimen group using MATLAB R2026a.

*E_r_*(*t*) = *a*_3_*t*^3^ + *b*_3_*t*^2^ + *c*_3_*t* + *d*_3_ (7)

where *t* is the exposure time (months); *a*_3_, *b*_3_, *c*_3_, and *d*_3_ are fitting parameters.

Table 9 summarizes the fitted equations and *R*^2^ for the *E_r_* vs. *t* for all specimen groups.

As shown in Table 9, the *R*^2^ for the cubic polynomial models of *E_r_* for all specimens exceeded 0.9910, indicating an excellent fit. *E_r_* = 60% was adopted as an analytical deterioration threshold with reference to the relative dynamic-modulus criteria used in GB/T 50082-2024 [55]. Its application to the present natural saline-soil exposure is an extrapolative modeling choice and should not be interpreted as a directly validated field failure criterion. The resulting crossing times are therefore treated as exploratory indicators.

**Figure 14:**
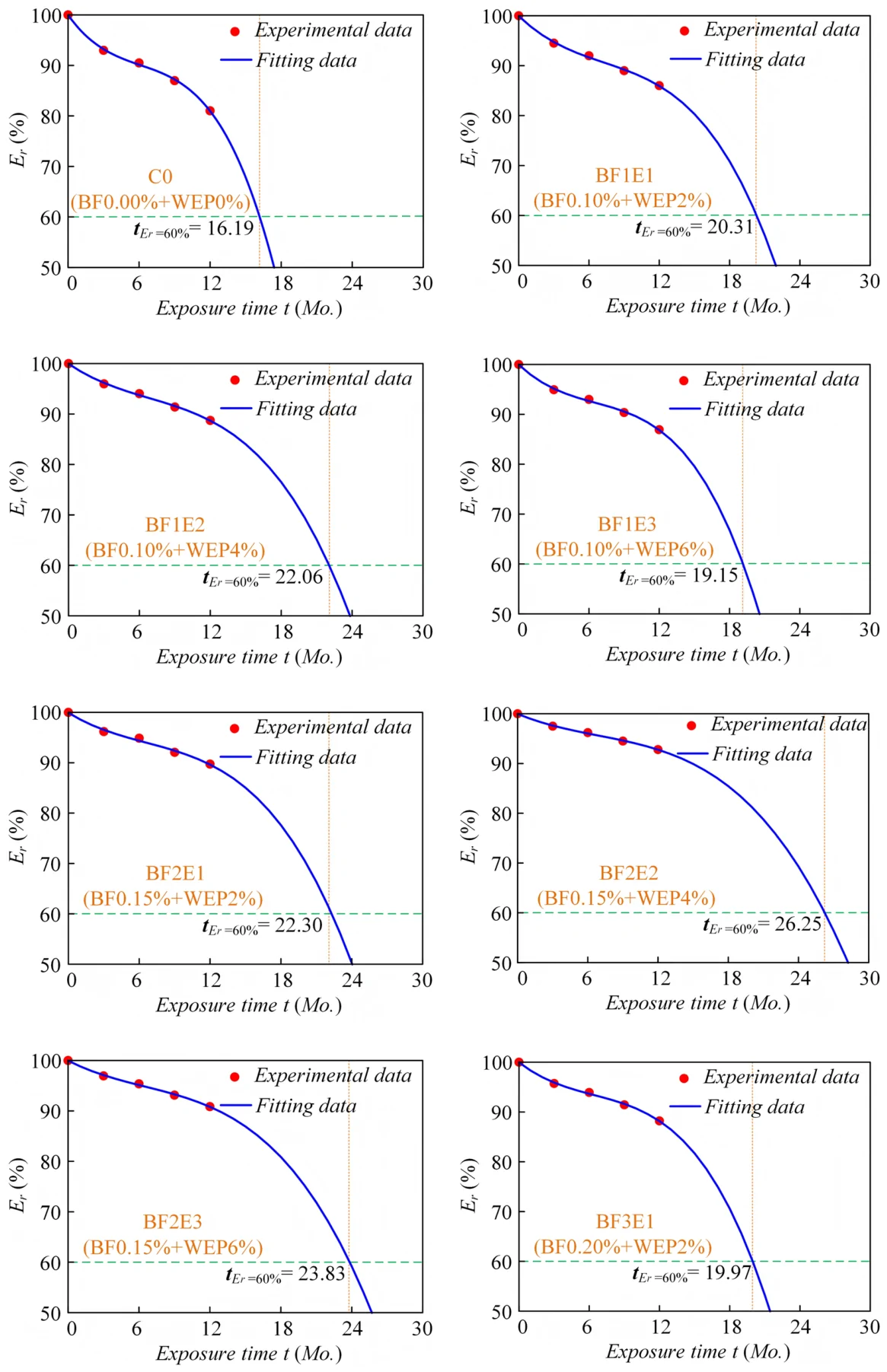

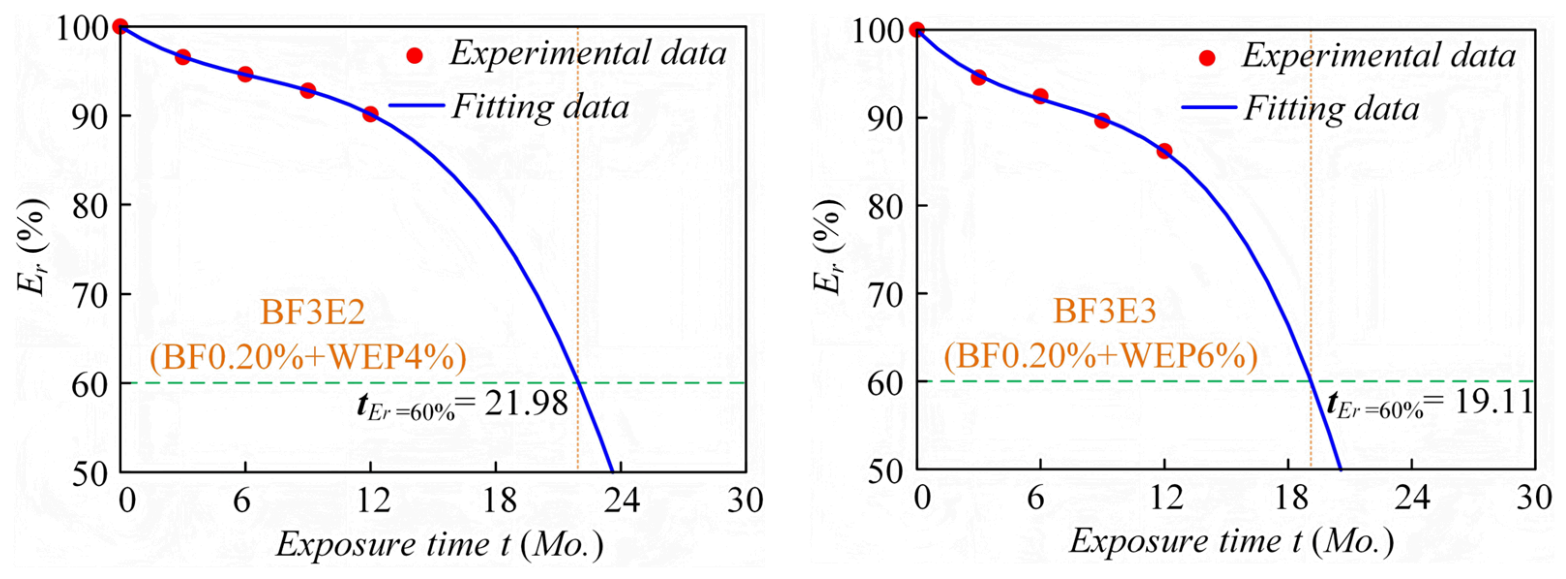
Cubic fits of *E_r_* over the measured interval and exploratory extrapolation to the adopted threshold.

Substituting *E_r_* = 60% into the fitted equation, the corresponding exposure time (*t*) was calculated as the *ETT* of the concrete. The fitted curves for *E_r_* and the *ETT* for all specimens are shown in Figure 14.

### 4.4. Service Life Prediction Results

Based on the cubic polynomial damage evolution equations, the exploratory *ETT*s for the ten concrete mixtures are summarized in Table 10.

Table 10 presents the following findings:

The *ETT* of all ten concrete mixtures was determined by the *E_r_* criterion. The BF2E2 mixture demonstrated the longest *ETT*, with a 62.14% increase compared to C0, followed by BF2E3 at 47.19%. Considering both engineering economy and durability performance, BF2E2 exhibited the most favorable overall performance.

With a fixed WEP dosage of 4%, comparison of BF1E2, BF2E2, and BF3E2 indicates that the *ETT* increased slightly as the BF dosage rose from 0.10% to 0.15%. However, when the BF dosage reached 0.20%, the *ETT* decreased. This outcome is attributed to an appropriate BF dosage that forms a uniform three-dimensional network within the concrete matrix, thereby suppressing microcrack initiation and propagation. In contrast, excessive BF tends to agglomerate, introducing initial defects and accelerating damage evolution.

With a fixed BF dosage of 0.15%, comparison of BF2E1, BF2E2, and BF2E3 shows that the *ETT* progressively increased as the WEP dosage rose from 2% to 4%. However, when the WEP dosage reached 6%, the *ETT* decreased. This result is due to an appropriate WEP dosage, which fills internal pores and improves interfacial bonding strength, while excessive WEP coats some aggregates, impeding hydration and reducing compactness.

## 5. Mechanistic Analysis

### 5.1. Deterioration Mechanism of Concrete in Saline Environment

#### 5.1.1. Sulfate Attack Mechanism

**Figure 15:**
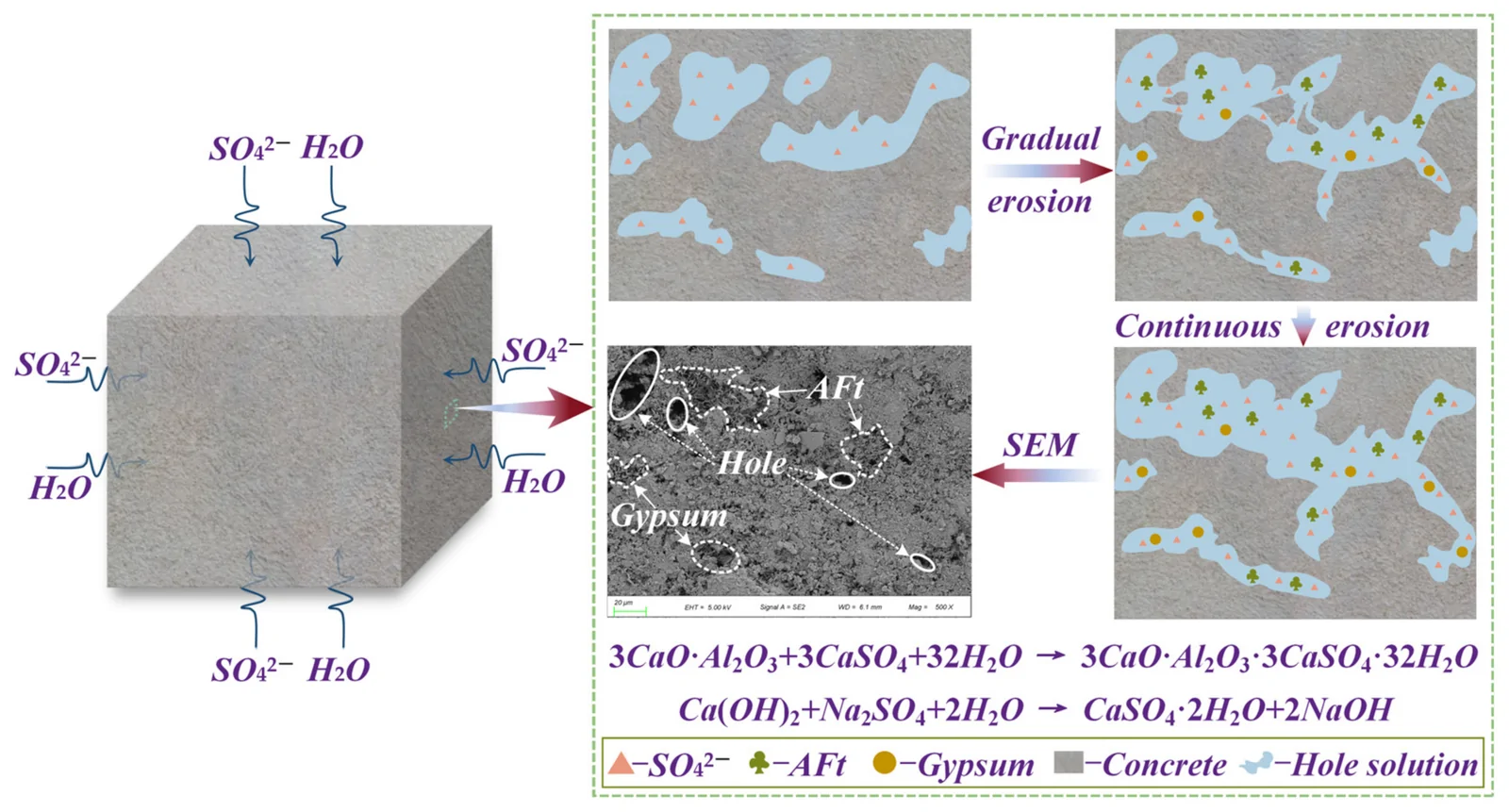
Schematic illustration of the sulfate attack mechanism of concrete.

Sulfate ions (SO_4_^2−^) present in saline environments react with cement hydration products to form expansive compounds, particularly AFt and gypsum. The development of these phases generates internal expansive stresses within the concrete microstructure, leading to cracking and subsequent deterioration [57,58,59,60]. The mechanism of sulfate attack is depicted in Figure 15.

High-intensity diffraction peaks of AFt (2θ = 36.1°) and gypsum (2θ = 27.5°) were detected in the XRD pattern of group C0, SEM observation revealed the formation of AFt and gypsum within pores and cracks. These indicated the extensive progression of these corrosion products.

#### 5.1.2. Chloride Attack Mechanism

**Figure 16:**
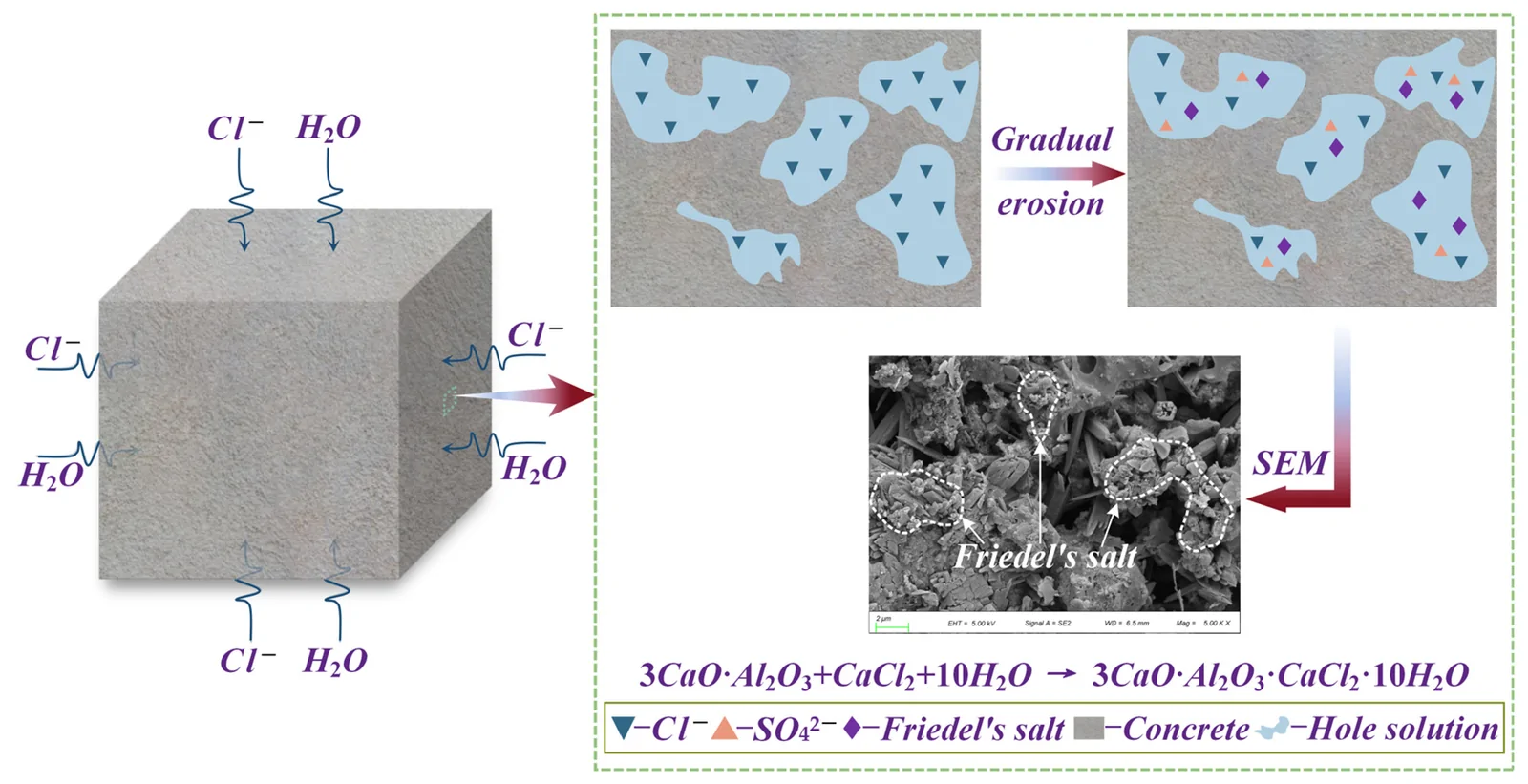
Schematic illustration of the chloride attack mechanism of concrete.

Chloride ions (*Cl*^−^) migrate into the concrete interior through the pore solution and react with cement hydration products to form Friedel’s salt [60,61]. The mechanism of chloride-induced corrosion is illustrated in Figure 16.

This erosion reaction consumes hydration products and alters their stability. Distinctive characteristic diffraction peaks of Friedel’s salt were detected at 2θ = 20.9° and 77.6° in the XRD pattern of group C0, SEM observation revealed the formation of Friedel’s salt within the concrete, indicating the occurrence of the aforementioned reaction.

#### 5.1.3. Combined Effect of Sulfate-Chloride Attack

In saline environments, SO_4_^2−^ and Cl^−^ ions exert a combined deterioration effect on concrete [60]. The presence of Cl^−^ promotes the formation of Friedel’s salt, which consumes 3CaO·Al_2_O_3_. SO_4_^2−^ promotes gypsum formation, thereby increasing expansive stresses. The simultaneous action of these aggressive agents accelerates concrete deterioration. After 12 months, the Δ*M* of C0 reached 1.720%, Δ*f* reached 13.52%, and *E_r_* decreased to 81.01%, collectively indicating the severity of the combined attack.

### 5.2. Reinforcement Mechanism of Composite Modification

#### 5.2.1. Reinforcement Mechanism of BF

**Figure 17:**
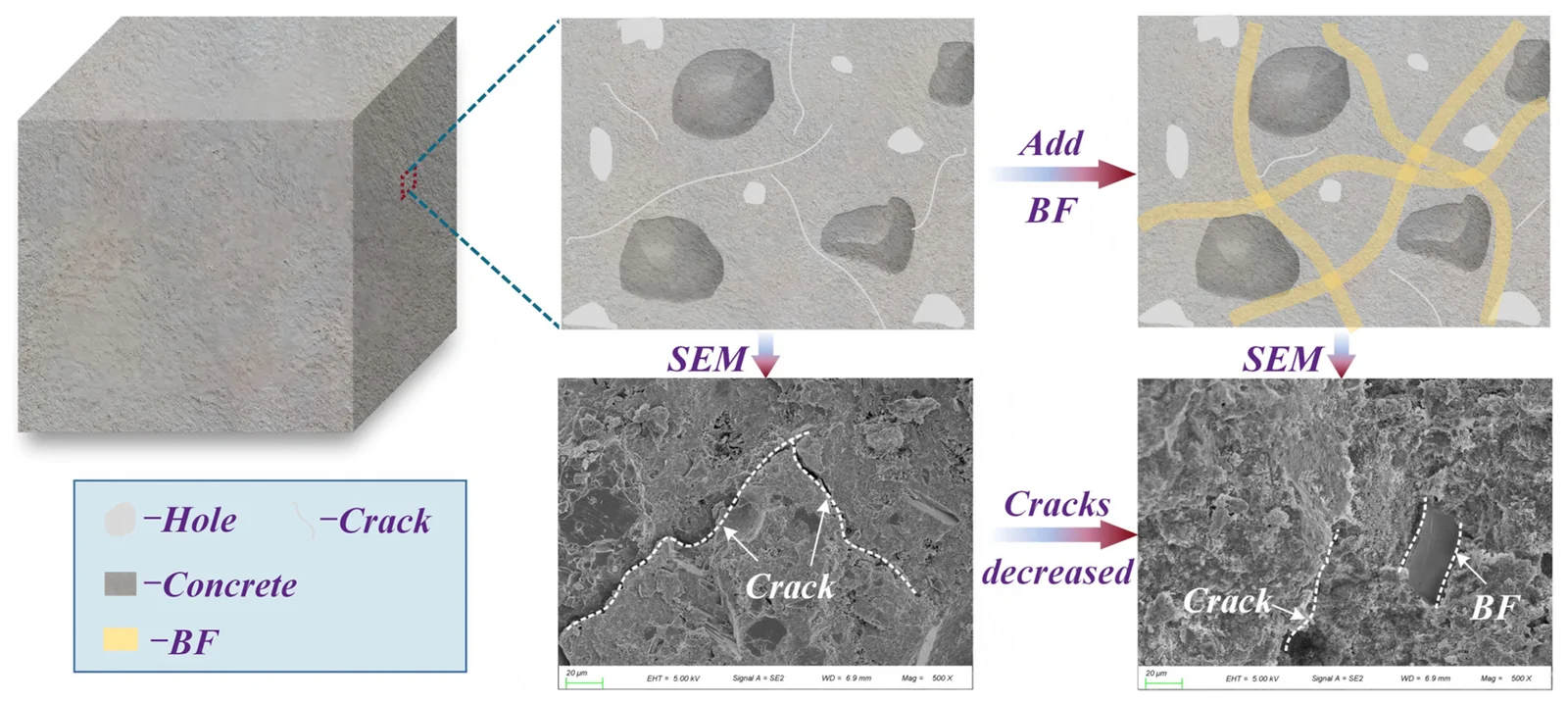
Schematic illustration of the reinforcement mechanism of BF in concrete: crack bridging and suppression of crack propagation.

Figure 17 illustrates the reinforcement mechanism of BF. The fibers are randomly oriented in three dimensions within the concrete matrix, forming a network structure that operates primarily through a fiber-bridging mechanism. When microcracks develop due to saline erosion, the BF filaments spanning these cracks transfer stress via fiber-matrix interfacial bonding, which restrains further crack propagation. SEM observations reflected that the crack width in the BF2E2 group decreased from 7–32 μm in C0 to below 7 μm, and the cracks changed from an interconnected open configuration to isolated closed states. These findings provide strong evidence for the effectiveness of the bridging mechanism.

#### 5.2.2. Modification Mechanism of WEP

**Figure 18:**
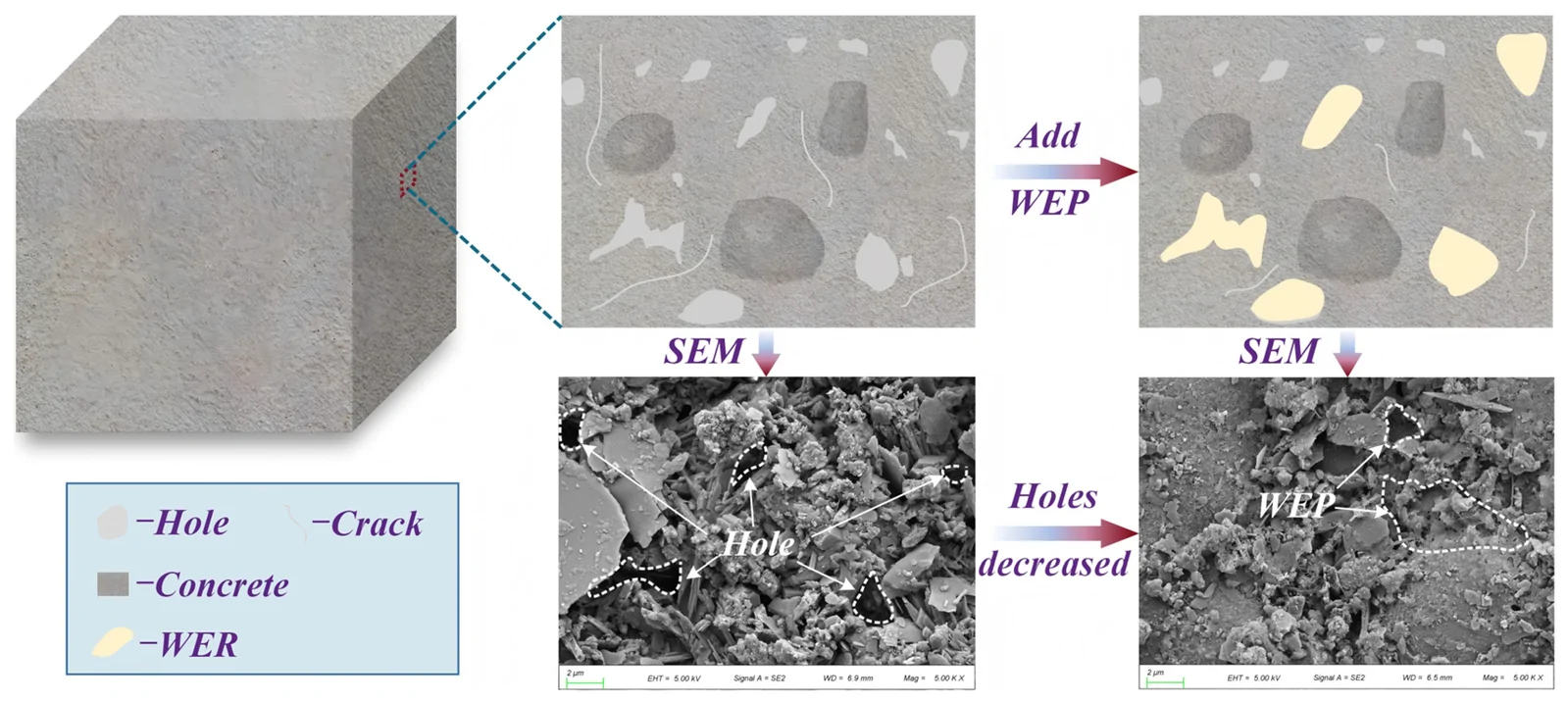
Schematic illustration of the modification mechanism of WEP in concrete: hole filling.

The modification mechanism of WEP is depicted in Figure 18. WEP operates via two primary mechanisms: pore filling and interfacial enhancement within the concrete matrix. During hydration, WEP particles occupy capillary pores, forming a dense, hydrophobic film that significantly reduces pore connectivity. Experimental results demonstrate a 28.64% reduction in total porosity and a 36.67% decrease in average pore area for BF2E2. XRD analysis indicates that the diffraction peak intensities of ettringite, gypsum, and Friedel’s salt in BF2E2 are substantially reduced, thereby inhibiting corrosion reactions at origin.

#### 5.2.3. Combined Enhancement Effect of BF-WEP

**Figure 19:**
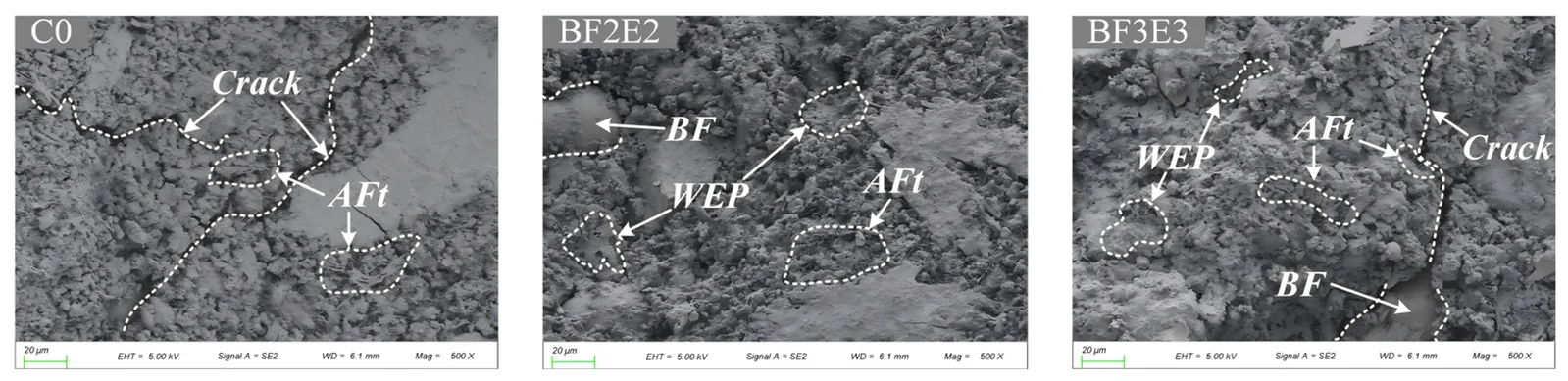
Microstructural morphology of concrete after 12 months of exposure.

The three-dimensional BF skeleton provides mechanical support and crack control, while the WEP polymer film fills pores and blocks transport pathways for aggressive agents. The two modifiers function collaboratively through a dual reinforcement network of “fiber bridging–polymer filling”. As shown in Figure 19, when the BF dosage exceeded 0.15% or the WEP dosage exceeded 4%, fiber agglomeration or emulsion-coated aggregates caused a rebound in porosity and an increase in cracks and corrosion products. It should be noted that the absence of BF-only and WEP-only control mixtures precludes a definitive demonstration of synergy in the strict sense (i.e., an effect greater than the sum of individual effects). The observed benefits may reflect additive contributions from fiber bridging and polymer filling rather than a true combined interaction.

## 6. Conclusions

This study modified concrete by incorporating both BF and WEP. A one-year outdoor natural exposure test systematically investigated the effects of varying dosages on durability performance, microstructure, and damage evolution. The main conclusions are summarized as follows:

(1) Relative to C0, the BF2E2 mixture achieved a 54.65% reduction in Δ*M*, a 37.20% reduction in Δ*f*, and a 14.55% increase in *E_r_*. Total porosity decreased by 28.64%, average pore area decreased by 36.67%, and crack width was reduced from 7–32 μm to below 7 μm. These results demonstrate the superior durability performance of the BF2E2 mixture under saline soil exposure.
(2) Microstructural analysis showed that the modified specimens have a denser matrix and significantly reduced diffraction peak intensities of corrosion products. This indicates that the combined modification optimizes the micro-pore structure of cement-based materials and establishes a dual physical and chemical protection system.
(3) The *ETT* predictions using damage evolution models indicated that the *ETT* of BF2E2 increased by 62.14% compared to C0. The *R*^2^ values for all groups exceeded 0.9880, indicating model reliability and supporting the theoretical basis for durability design of modified concrete. The *E_r_* criterion governed the *ETT* of all specimens and can be monitored in real time using non-destructive testing methods.
(4) Mechanistic analysis revealed that BF and WEP function collaboratively through crack bridging and pore filling, forming a dual reinforcement network of “fiber bridging–polymer filling” that enhances the durability of concrete under saline soil exposure.

## Figures and Tables

**Table 1:** Technical specifications of WEP.

Type	Appearance	Solid Content/%	PH	Epoxy Value	Viscosity/Pa·s	Density/kg·m^−3^
DY-128-50	Milky-white homogeneous liquid	50 ± 3	6~8	0.52	≤2	1050

**Table 2:** Technical specifications of curing agent.

Type	Appearance	Solid Content/%	PH	Viscosity/Pa·s	Amine Value
DY-175	Light-brown homogeneous liquid	49~51	9.5~10.5	6.5~9	330

**Table 3:** Technical specifications of defoamer.

Type	Appearance	PH	Defoaming Speed/mL·s^−1^
LJ-618	Milky-white water-based liquid	6~8	≥45

**Table 4:** Physical and mechanical properties of BF.

Monofilament Diameter/μm	Length/mm	Density/kg·m^−3^	Tensile Strength/MPa	Elastic Modulus/GPa	Ultimate Elongation/%
30	18	2699	≥2000	≥85	≥2.5

**Table 5:** Mix proportions of concrete specimens.

Specimen No.	Specimen ID	Cement/kg·m^−3^	Sand/kg·m^−3^	Gravel/kg·m^−3^	Water/kg·m^−3^	BF/kg·m^−3^	WEP/kg·m^−3^	Curing Agent/kg·m^−3^	Defoamer/kg·m^−3^
1	C0	342	704	1149	205	0	0	0	0
2	BF1E1	342	704	1149	205	2.699	6.84	2.28	0.0342
3	BF1E2	342	704	1149	205	2.699	13.68	4.56	0.0684
4	BF1E3	342	704	1149	205	2.699	20.52	6.84	0.1026
5	BF2E1	342	704	1149	205	4.049	6.84	2.28	0.0342
6	BF2E2	342	704	1149	205	4.049	13.68	4.56	0.0684
7	BF2E3	342	704	1149	205	4.049	20.52	6.84	0.1026
8	BF3E1	342	704	1149	205	5.398	6.84	2.28	0.0342
9	BF3E2	342	704	1149	205	5.398	13.68	4.56	0.0684
10	BF3E3	342	704	1149	205	5.398	20.52	6.84	0.1026

**Table 6:** Image-based pore feature analysis of specimens after 12 months of exposure.

Specimen No.	Specimen ID	Number of Pores	Total Pore Area/μm^2^	Average Pore Area/μm^2^	Porosity/%
1	C0	20,409	7350.926	0.360	18.928
2	BF1E1	27,637	7135.926	0.258	18.374
3	BF1E2	19,418	5618.679	0.289	14.468
4	BF1E3	16,127	6338.593	0.393	16.321
5	BF2E1	17,291	5462.914	0.316	14.067
6	BF2E2	23,046	5245.691	0.228	13.507
7	BF2E3	16,876	5458.506	0.323	14.055
8	BF3E1	20,313	6595.012	0.325	16.982
9	BF3E2	21,501	5955.864	0.277	15.336
10	BF3E3	20,429	6681.173	0.327	17.203

**Table 7:** Fitted equations for Δ*M* vs. *t*.

Specimen ID	Fitted Equation	*R* ^2^
C0	Δ*M*(*t*) = 5.4086 × 10^−4^*t*^3^ − 7.5783 × 10^−3^*t*^2^ + 1.5641 × 10^−1^*t* − 7.3058 × 10^−3^	0.9979
BF1E1	Δ*M*(*t*) = 2.5010 × 10^−4^*t*^3^ − 4.3060 × 10^−3^*t*^2^ + 1.3456 × 10^−1^*t* − 8.1032 × 10^−3^	0.9963
BF1E2	Δ*M*(*t*) = 4.4751 × 10^−4^*t*^3^ − 7.6062 × 10^−3^*t*^2^ + 1.3322 × 10^−1^*t* − 1.2781 × 10^−2^	0.9884
BF1E3	Δ*M*(*t*) = 4.2080 × 10^−4^*t*^3^ − 7.0473 × 10^−3^*t*^2^ + 1.4196 × 10^−1^*t* − 1.0438 × 10^−2^	0.9937
BF2E1	Δ*M*(*t*) = 1.9413 × 10^−4^*t*^3^ − 2.9119 × 10^−3^*t*^2^ + 1.0716 × 10^−1^*t* − 4.1932 × 10^−3^	0.9986
BF2E2	Δ*M*(*t*) = 1.4041 × 10^−4^*t*^3^ − 1.9997 × 10^−3^*t*^2^ + 6.8808 × 10^−2^*t* − 2.0497 × 10^−3^	0.9992
BF2E3	Δ*M*(*t*) = 2.0502 × 10^−4^*t*^3^ − 3.3574 × 10^−3^*t*^2^ + 9.4382 × 10^−2^*t* − 2.7470 × 10^−3^	0.9991
BF3E1	Δ*M*(*t*) = 4.2393 × 10^−4^*t*^3^ − 7.2115 × 10^−3^*t*^2^ + 1.3731 × 10^−1^*t* − 1.1119 × 10^−2^	0.9921
BF3E2	Δ*M*(*t*) = 4.3039 × 10^−4^*t*^3^ − 6.9170 × 10^−3^*t*^2^ + 1.2096 × 10^−1^*t* − 6.6735 × 10^−3^	0.9964
BF3E3	Δ*M*(*t*) = 1.4954 × 10^−4^*t*^3^ − 1.8951 × 10^−3^*t*^2^ + 1.2464 × 10^−1^*t* − 5.7349 × 10^−3^	0.9983

**Table 8:** Fitted equations for Δ*f* vs. *t*.

Specimen ID	Fitted Equation	*R* ^2^
C0	Δ*f*(*t*) = 6.7668 × 10^−3^*t*^3^ − 1.3775 × 10^−1^*t*^2^ + 1.8053*t* + 2.9581 × 10^−2^	0.9994
BF1E1	Δ*f*(*t*) = 4.7161 × 10^−3^*t*^3^ − 1.0914 × 10^−1^*t*^2^ + 1.5880*t* + 2.1879 × 10^−2^	0.9996
BF1E2	Δ*f*(*t*) = 3.4199 × 10^−3^*t*^3^ − 8.1344 × 10^−2^*t*^2^ + 1.3601*t* + 9.4974 × 10^−3^	0.9999
BF1E3	Δ*f*(*t*) = 5.0567 × 10^−3^*t*^3^ − 1.1563 × 10^−1^*t*^2^ + 1.6444*t* + 1.3375 × 10^−2^	0.9998
BF2E1	Δ*f*(*t*) = 2.4848 × 10^−3^*t*^3^ − 6.1155 × 10^−2^*t*^2^ + 1.2099*t* + 1.4787 × 10^−2^	0.9997
BF2E2	Δ*f*(*t*) = 1.5212 × 10^−3^*t*^3^ − 3.9987 × 10^−2^*t*^2^ + 9.6816 × 10^−1^*t* + 4.1465 × 10^−2^	0.9973
BF2E3	Δ*f*(*t*) = 2.4207 × 10^−3^*t*^3^ − 5.8246 × 10^−2^*t*^2^ + 1.1160*t* + 2.7211 × 10^−2^	0.9990
BF3E1	Δ*f*(*t*) = 4.1392 × 10^−3^*t*^3^ − 9.9033 × 10^−2^*t*^2^ + 1.4913*t* +1.9159 × 10^−2^	0.9996
BF3E2	Δ*f*(*t*) = 2.2415 × 10^−3^*t*^3^ − 6.1186 × 10^−2^*t*^2^ + 1.2262*t* + 2.1548 × 10^−2^	0.9994
BF3E3	Δ*f*(*t*) = 3.7186 × 10^−3^*t*^3^ − 9.6987 × 10^−2^*t*^2^ + 1.5894*t* + 4.0980 × 10^−3^	0.9999

**Table 9:** Fitted equations for *E_r_* vs. *t*.

Specimen ID	Fitted Equation	*R* ^2^
C0	*E_r_*(*t*)= −2.1596 × 10^−2^*t*^3^ + 3.9718 × 10^−1^*t*^2^ − 3.2389*t* + 9.9943 × 10	0.9989
BF1E1	*E_r_*(*t*)= −9.2337 × 10^−3^*t*^3^ + 2.0207 × 10^−1^*t*^2^ − 2.2619*t* + 9.9943 × 10	0.9980
BF1E2	*E_r_*(*t*)= −6.4029 × 10^−3^*t*^3^ + 1.3144 × 10^−1^*t*^2^ − 1.5944*t* + 9.9951 × 10	0.9977
BF1E3	*E_r_*(*t*)= −1.2123 × 10^−2^*t*^3^ + 2.3860 × 10^−1^*t*^2^ − 2.2079*t* + 9.9948 × 10	0.9980
BF2E1	*E_r_*(*t*)= −6.2582 × 10^−3^*t*^3^ + 1.2415 × 10^−1^*t*^2^ − 1.4457*t* + 9.9915 × 10	0.9918
BF2E2	*E_r_*(*t*)= −3.6709 × 10^−3^*t*^3^ + 7.5609 × 10^−2^*t*^2^ − 9.7842 × 10^−1^*t* + 9.9971 × 10	0.9980
BF2E3	*E_r_*(*t*)= −4.7406 × 10^−3^*t*^3^ + 9.2501 × 10^−2^*t*^2^ − 1.1898*t* + 9.9961 × 10	0.9978
BF3E1	*E_r_*(*t*)= −9.9592 × 10^−3^*t*^3^ + 1.9048 × 10^−1^*t*^2^ − 1.8341*t* + 9.9956 × 10	0.9983
BF3E2	*E_r_*(*t*)= −7.1017 × 10^−3^*t*^3^ + 1.4116 × 10^−1^*t*^2^ − 1.4918*t* + 9.9993 × 10	0.9999
BF3E3	*E_r_*(*t*)= −1.2111 × 10^−2^*t*^3^ + 2.4444 × 10^−1^*t*^2^ − 2.3391*t* + 9.9944 × 10	0.9979

**Table 10:** Exploratory *ETT* for the ten mixtures.

Specimen ID	Time to Reach 5% Δ*M*/Months	Time to Reach 30% Δ*f*/Months	Time to Reach 60% *E_r_*/Months	*ETT*/Months	Equivalent Time/Years	Relative Difference vs. C0/%
C0	21.10	18.74	16.19	16.19	1.35	—
BF1E1	26.06	21.33	20.31	20.31	1.69	25.45
BF1E2	24.02	23.04	22.06	22.06	1.84	36.26
BF1E3	23.71	20.89	19.15	19.15	1.60	18.28
BF2E1	28.06	24.69	22.30	22.30	1.86	37.74
BF2E2	32.65	28.34	26.25	26.25	2.19	62.14
BF2E3	29.21	25.24	23.83	23.83	1.99	47.19
BF3E1	24.02	22.32	19.97	19.97	1.66	23.35
BF3E2	24.26	26.02	21.98	21.98	1.83	35.76
BF3E3	27.22	22.83	19.11	19.11	1.59	18.04

## Data Availability

The data presented in this study are available on request from the corresponding authors.
